# The roles of neuroticism, genetic susceptibility, and amygdala structure in chronic musculoskeletal pain onset and recovery

**DOI:** 10.1038/s43856-026-01754-9

**Published:** 2026-07-06

**Authors:** Mattia Cannistra, Yu Liu, Valeria Sacca, Sierra Hodges, Lynn Pham, Pierangelo Veltri, Phil Hyoun Lee, Jian Kong

**Affiliations:** 1 Department of Psychiatry, Massachusetts General Hospital and Harvard Medical School, Charlestown, MA USA; 2 Department of Computer, Modeling, Electronics and System Engineering, University of Calabria, Cosenza, Italy; 3 Psychiatric and Neurodevelopmental Genetics Unit, Center for Genomic Medicine, Massachusetts General Hospital, Boston, MA USA

**Keywords:** Genetics, Psychiatric disorders

## Abstract

**Background:**

Chronic musculoskeletal pain (CMP) represents a significant global health burden, yet the neurobiological mechanisms underlying its development remain poorly understood. This study investigates the complex interplay between neuroticism, genetic risk, and amygdala morphology in CMP onset, recovery, and pain characteristics using UK Biobank data.

**Methods:**

We conducted longitudinal analyses on (1) 9382 pain-free individuals followed for CMP development and (2) 3460 CMP patients tracked for recovery outcomes. Neuroticism was quantified via a validated 12-item questionnaire assessing psychological distress. To assess chronic pain trajectories, we examined polygenic risk scores (PRS) for CMP and neuroticism, along with amygdala grey matter volumes, as predictors. Multivariate logistic and linear regression models were employed, followed by mediation analyses to elucidate underlying relationships between variables. Analyses were restricted to individuals of European ancestry.

**Results:**

We found that higher neuroticism scores, neuroticism PRS, and CMP PRS were significantly associated with increased risk of CMP onset and reduced likelihood of recovery across cohorts (all *P*_*corrected*_ < 0.05). Both neuroticism scores and PRS were positively correlated with pain intensity, whereas CMP PRS were not. Additionally, amygdala grey matter volumes were significantly associated with neuroticism in the CMP onset cohort but not in the recovery group. Neuroticism mediated the relationship between CMP PRS and subsequent CMP onset with a small effect size, while no mediation was observed for CMP recovery.

**Conclusions:**

This study demonstrated significant associations between neuroticism, genetic factors, and amygdala morphology in CMP trajectories, highlighting a potential neurobiological mechanism linking psychological vulnerability to chronic pain. These results underscore the importance of integrating psychological assessment and targeted interventions into comprehensive pain management strategies.

## Plain Language Summary

Chronic musculoskeletal pain is a widespread condition that significantly impacts quality of life, yet the reasons why some people develop it or recover remain unclear. In this study, we followed participants over time to investigate how personality traits, genetic factors, and brain structure influence pain outcomes. We focused on neuroticism, a personality trait reflecting a tendency to experience negative emotions such as worry, stress, and emotional instability, genetic risk scores related to pain and neuroticism, and the amygdala, a brain region involved in emotional regulation. Individuals with higher neuroticism and genetic risk were more likely to develop chronic pain, experience persistent symptoms, and report greater pain intensity. Neuroticism partially explained the link between genetic risk and pain onset and was associated with larger amygdala grey matter volume. These findings underscore psychological and biological factors that may help identify at-risk individuals and improve prevention and treatment strategies.

## Abstract (web summary)

Cannistrà et al. analyze UKBiobank data to study how neuroticism, genetic risk, and amygdala structure influence chronic musculoskeletal pain outcomes. Higher neuroticism and genetic risk increase pain onset, contribute to persistent and intense pain, and correlate with amygdala volume, with neuroticism partly mediating genetic risk effects.

## Introduction

Chronic musculoskeletal pain (CMP) is a prevalent condition and a major contributor to disability, significantly diminishing quality of life and imposing considerable socioeconomic burdens^1,2^. The treatment of CMP remains unsatisfactory, highlighting the need for a deeper understanding of its risk factors and underlying neurobiological mechanisms^3^.

Neuroticism, a personality trait marked by emotional instability and a predisposition to experiencing negative emotions such as anxiety and worry, has emerged as a key determinant of various health outcomes^4^. Recent research suggests that high neuroticism is linked to worse pain perception and the persistence of chronic pain^5,6^. Individuals with high neuroticism tend to experience heightened sensitivity to pain, increased pain-related anxiety, and a greater tendency to catastrophize, which can contribute to both the intensity and persistence of chronic musculoskeletal pain^5–7^. Conversely, positive psychological states may help mitigate the impact of pain^8,9^. In parallel, chronic pain is frequently observed in individuals with neuropsychiatric disorders, further highlighting the bidirectional relationship between negative mental health states, such as neuroticism, and the occurrence of chronic pain^10^.

Notably, this relationship appears to extend beyond psychological processes, with growing evidence supporting shared biological mechanisms. In fact, mental health traits such as neuroticism and depression have been shown to share common genetic risk factors with chronic pain conditions, emphasizing the role of overlapping biological pathways in their co-occurrence^11,12^. Genetic studies have uncovered variants that affect chronic pain risk via interactions with neuroticism. Broader analyses also indicate that many loci influencing CMP are enriched in central nervous system pathways and are genetically correlated with neuroticism and related psychiatric traits^13,14^. These convergent biological mechanisms may simultaneously increase susceptibility to neuroticism and CMP^13,14^.

A key brain region influenced by these shared pathways is the amygdala, which is central to emotional processing and the brain’s response to stress and affective stimuli. Previous research suggests that both high neuroticism and chronic pain contribute to functional and structural changes within this region^15,16^. In particular, individuals with a high level of neuroticism are associated with increased grey matter volumes in the amygdala^17^ and strengthened functional connectivity between the amygdala and precuneus^18^. Similarly, chronic pain patients also exhibit increased grey matter volume, particularly in the central and cortical nuclei of the amygdala^19^, along with enhanced functional connectivity between the amygdala and medial prefrontal cortex, as well as other pain- and emotion-related regions^20^.

Despite evidence linking neuroticism and chronic pain, there remains a lack of longitudinal analyses that offer a deeper understanding of these dynamics over time, thereby limiting causal inference. Moreover, although neuroticism may influence emotional responses to pain by affecting areas like the amygdala, there is limited direct evidence linking their interplay specifically to CMP trajectories. Few previous studies have examined this relationship prospectively in pain-free populations, which limits causal inference about the directionality of the link between neuroticism and CMP.

Thus, integrating these variables at both phenotypic and biological levels may enhance the ability to identify individuals at risk of developing CMP, offering a more comprehensive approach to early detection and prevention. These factors may also provide insight into the likelihood of CMP recovery, supporting more targeted and personalized pain management strategies. In this context, polygenic risk scores (PRS), calculated by aggregating the effects of genetic variants linked to specific conditions across the genome, may serve as a key factor for the identification of individuals with varying levels of genetic susceptibility^21,22^. This approach has been shown to be highly valuable for gaining insight into the biological foundations of an increased risk of developing certain conditions^23^.

By leveraging the extensive UK Biobank dataset, this study aims to investigate the complex interplay between neuroticism, amygdala structure, and genetic risk in CMP onset and recovery. We conducted longitudinal analyses focused on the following objectives: (1) to investigate whether neuroticism phenotypes, along with CMP and neuroticism PRSs, serve as risk factors for the onset and recovery of CMP across multiple timepoints, (2) to examine the interplay of genetic susceptibility and neuroticism as potential contributors to CMP onset and recovery, and (3) to explore the relationship between neuroticism and amygdala grey matter volumes in CMP onset and recovery. Additionally, we assessed the extent to which genetic risk and neuroticism exacerbate pain characteristics, including pain intensity and the number of pain sites.

We show that higher neuroticism and greater genetic risk increase the likelihood of developing chronic pain and contribute to persistent and more intense pain. Neuroticism partly mediates the effect of genetic predisposition on pain onset and is associated with increased amygdala volume. In a longitudinal analysis, we examine how these psychological, genetic, and neurobiological factors interact to shape pain outcomes over time. These findings provide insights into the mechanisms underlying chronic pain and highlight potential targets for early identification, prevention, and personalized management strategies.

## Methods and Materials

### Ethics Statement

This study used data from the UK Biobank under approved application #32568. UK Biobank has ethical approval from the North West Multi-centre Research Ethics Committee (reference numbers 06/MRE08/65, 11/NW/0382, and 16/NW/0274). All participants provided informed consent for their data to be used for health-related research.

### Study population

The dataset for this study was derived from the UK Biobank (https://www.ukbiobank.ac.uk) cohort^24^, which included over 500,000 participants aged 40-69 years, recruited across Wales, England, and Scotland from 2006 to 2019 + . All participants provided informed consent for access to their medical records and health information for research purposes. The UK Biobank cohort provides longitudinal data with different timepoints: first assessment – timepoint 1 (TP1) (2006–2010), second assessment – TP2 (2012–2013), Imaging visit 1 – TP3 (2014 + ), Imaging visit 2 – TP4 (2019 + ), and Pain Questionnaire – PQ (2019) assessment. Additionally, genome-wide genetic data are available for over 488,000 participants.

By using longitudinal data at different timepoints, we performed two separate analyses on (i) participants who reported no pain at baseline but later developed CMP at a subsequent timepoint and (ii) those who experienced CMP at baseline but later recovered from the condition.

**Fig. 1:**
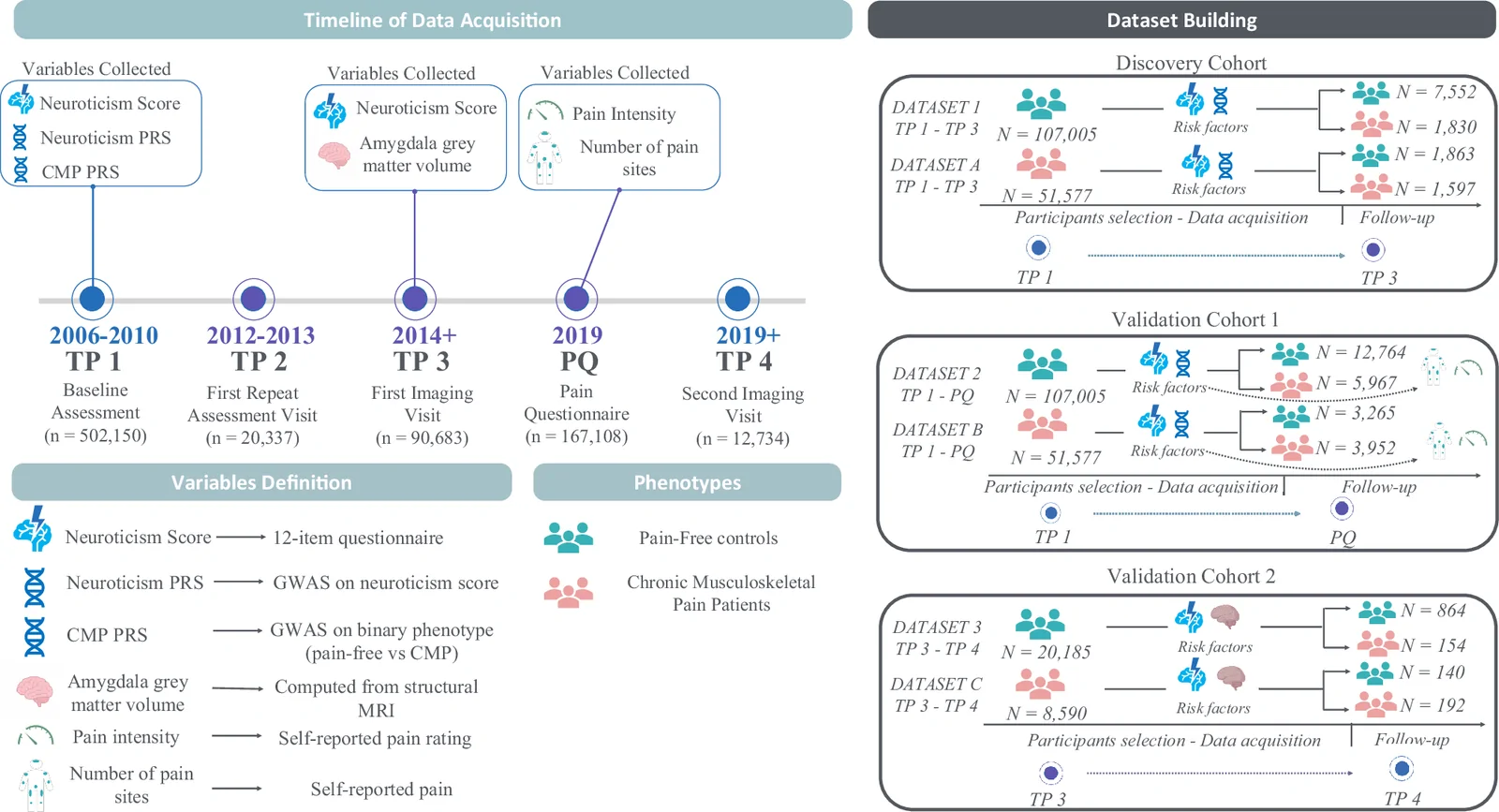
Overview of the study design. The variables used in the study were collected across multiple assessments (top left). A longitudinal analysis was conducted to track chronic musculoskeletal pain (CMP) onset in a cohort of healthy individuals. Similarly, participants with a CMP at baseline were followed over time to track their recovery. Neuroticism was estimated using a 12-item questionnaire, and two distinct polygenic risk scores (PRSs) were derived from GWASs on neuroticism and CMP phenotypes. Amygdala grey matter volume and pain characteristics were additionally computed for all individuals included in the final cohorts (bottom left). These variables were evaluated as risk factors for CMP onset and recovery, and their associations with pain characteristics were assessed. A discovery and two validation cohorts were constructed (right panels).

The primary analysis in this study examined the influence of neuroticism and neuroticism/CMP PRSs on the onset of CMP by comparing a group of pain-free participants who remained pain-free at a subsequent timepoint with those who were pain-free at baseline but later developed a CMP condition. In a parallel analysis, we explored the role of neuroticism and neuroticism/CMP PRSs on the recovery from CMP by comparing a group of individuals with CMP who later recovered to those who experienced persistent (not recovered) CMP across timepoints. For both analyses, we constructed distinct discovery and validation cohorts, ensuring they were independent by including each participant in only one cohort, with no overlap between them. Genetic analyses were restricted to individuals of European ancestry. We investigated (i) the association between neuroticism score, neuroticism PRS, and pain intensity phenotypes and (ii) the association between neuroticism score and amygdala grey matter volumes in both CMP onset and recovery analyses. The study design is illustrated in Fig. 1.

### Inclusion/Exclusion criteria

Prior to selecting participants to include in the different cohorts, exclusion criteria were applied to avoid potential confounds. Following previous studies^25–27^, participants were excluded if they had any psychological or neurological disorders defined by the International Classification of Diseases 10th (ICD-10) and 9th Revision (ICD-9). A list of the disorders used for exclusion can be found in Suppl. Table S1. Notably, the ICD-9/10 codes used for our exclusion criteria encompass relevant inflammatory and autoimmune musculoskeletal conditions (including Rheumatoid Arthritis and Ankylosing Spondylitis), which are common in clinical populations and may manifest as CMP. Despite the scope of these exclusion codes, we acknowledge that some residual heterogeneity may remain and influence our findings. Additionally, participants who reported long-term standing illness, disability or infirmity, or a diabetes diagnosis from a physician were excluded from the analysis.

### Pain-free and CMP phenotypes

At each TP, after applying exclusion criteria (Supplementary Methods), we first identified the pain-free and CMP participants using pain-related questions. To identify participants without any acute or chronic pain, individuals were asked, “In the last month, have you experienced any pain that interfered with your usual activities?” (field 6159). Respondents could select any pain among (i) headache, (ii) facial pain, (iii) abdominal pain, (iv) neck pain, (v) back pain, (vi) knee pain, (vii) hip pain, or (viii) pain all over the body, as well as ‘None of the above’ or ‘Prefer not to answer’ options. All participants who selected ‘Prefer not to answer’ were excluded from the analysis. Those who selected ‘None of the above’ were classified as pain-free. If any pain was reported in field 6159 (i.e., items i-viii), additional questions were asked to determine whether their pain had persisted for more than three months, indicating a chronic pain condition (fields 3571, 3414, 3773, 3404, 4067, 3741, 2956, 3799) (Suppl. Table S2).

To identify the CMP cohort at each TP, we identified subjects who reported at least one CMP in the back, hip, knee, or neck. Among these participants, those who reported other types of coexisting pain (headache, facial, or abdominal) were excluded from the analysis. Individuals who reported pain all over the body were also excluded, as their etiology in the UK Biobank was unknown and could reflect heterogeneous conditions such as fibromyalgia that risked introducing classification bias into the CMP cohort. At the PQ assessment, chronic pain status was determined using the question: “Are you troubled by pain or discomfort, either all the time or on and off, that has been present for more than 3 months?” (field 120019). Participants who reported no pain (‘No’ answers) were classified as pain-free. Similar to the other timepoints, only participants who reported a CMP and no coexisting non-musculoskeletal pain were included in the analysis. In this way, we extracted pain-free and CMP participants at each TP. We acknowledge that while the TP1–TP4 definition of pain-free excluded both chronic and past-month acute pain, the PQ definition relied on a single question, excluding pain present for over 3 months. While this method successfully identifies individuals with chronic pain, it has the potential to result in misclassification bias by delineating individuals with acute or subacute pain ( < 3 months) as controls. However, such bias would likely attenuate observed associations, making our findings conservative.

### CMP onset and recovery datasets

For the CMP onset analysis, we first extracted pain-free participants at TP1 and classified them into two sub-groups based on whether they remained pain-free (Group 1) or developed CMP (Group 2) at the follow-up assessment (TP3). This dataset represented the discovery cohort (Discovery cohort: Dataset 1, TP1–TP3). To construct the first validation cohort (Dataset 2: first validation cohort, TP1–PQ), pain-free participants extracted at TP1 were classified into two groups according to their follow-up (PQ assessment) condition (Group 1: steady pain-free, Group 2: CMP onset). Similarly, the second validation cohort (Dataset 3: second validation cohort, TP3–TP4) leveraged pain-free participants at TP3 as the baseline and TP4 as the follow-up (Fig. 1).

Similarly, to build datasets for the recovery analysis, we identified participants with CMP at baseline (TP1) and divided them into two groups based on whether they recovered (Group 1) or remained with CMP (Group 2) by the follow-up at TP3 (Discovery cohort: Dataset A, TP1–TP3). Consistent with the CMP onset dataset formation, the validation cohorts were built using the same baseline and follow-up timepoints: TP1 as the baseline and PQ as the follow-up assessment (Dataset B: first validation cohort, TP1–PQ) and TP3 to TP4 (Dataset C: second validation cohort, TP3–TP4) (Fig. 1).

TP1, TP3, and PQ included substantially larger numbers of participants, whereas TP2 was attended by only a small subset. For this reason, dataset construction focused on TP1, TP3, and PQ, which maximized sample size and analytic power while also allowing inclusion of brain imaging data at TP3. For longitudinal analyses with TP3 serving as the baseline (the first visit with imaging data), we used TP4 as the follow-up timepoint.

Across all cohorts, any participants with uninterpretable answers (i.e., ‘Do not know’ or ‘Prefer not to answer’), missing values in any variables and covariates used for analysis, or missing follow-up assessments, were excluded from the analysis. To maintain the independence of our datasets, any participants eligible for multiple cohorts were included in only a single cohort. The discovery cohorts (Dataset 1 and A) were given priority during the assignment process to preserve sample size and statistical power for the primary analysis. Participants assigned to the discovery cohorts were subsequently excluded from all validation cohorts, ensuring no overlap between the datasets. We acknowledge that this prioritization strategy may reduce the representativeness and statistical power of the validation cohorts and introduce selection bias. Flowcharts depicting dataset construction for the discovery cohorts in the CMP onset and recovery analyses can be found in Figs. S1, S2, while those for the validation cohorts can be found in Figs. S3 and S4. The programming language Python (v 3.7.3)^28^ was used for data extraction and cleaning.

Our exclusion criteria ensured that there were no overlapping participants across datasets, which accounted for the possibility of a change in pain condition from TP3 to PQ. However, we also examined any change in pain status at the intermediate TP2 assessment. In Dataset 1, we found 64 participants (0.6% of the sample) who were pain-free at TP1, CMP at TP2, and then pain-free again at TP3. These individuals were classified as steady pain-free in Dataset 1. Similarly, in Dataset A, we identified 35 participants (1.01% of the sample) who were CMP at TP1, pain-free at TP2, and CMP again at TP3. These participants were classified as persistent CMP in Dataset A. Given the small number of people with altered pain statuses at TP2, we do not believe these changes meaningfully affected the results.

### Neuroticism score

Neuroticism scores were calculated based on self-reported responses to twelve questions that assessed tendencies toward emotional instability, worry, and other related neurotic behaviors. The full list of the 12 items is provided in the Supplementary Methods (Table S2). Each question was coded as 0 for a ‘No’ response and 1 for a ‘Yes’ response. A composite neuroticism score was computed by summing across all questions using the validated Eysenck Personality Inventory Neuroticism scale (EPIN-R)^29^. The total score ranged from 0 to 12 (0 = lowest neurotic behavior, 12 = highest neurotic behavior), providing a measure of the participant’s level of neuroticism^30^.

### Pain intensity and pain sites phenotypes

Pain intensity and pain sites were defined based on the information extracted from the PQ assessment, as no other timepoint provided pain intensity ratings. At the PQ assessment, participants were asked to rate their pain in four musculoskeletal regions (neck and shoulder, back, hip, and knee) on a scale of 0-10. (fields 120025, 120026, 120028, 120029) (Suppl. Table S2). For individuals who reported pain in multiple regions, the worst pain intensity score was defined as the highest pain intensity rating across the four sites. For participants who reported only one pain, the worst pain intensity score corresponded to their single reported value.

To measure the cumulative pain burden across both the number of affected sites and intensity at each site, the total pain intensity score was calculated as the sum of the pain intensity ratings across all four pain sites, yielding a range of 1–40. The number of coexisting pains was established as the count of pain sites across the four areas. Any area with a score between 1 and 10 was considered a pain site, resulting in a value ranging from 1 to 4 (Suppl. Table S2).

### MRI data acquisition and preprocessing

During Imaging visit 1, participants underwent a structural MRI (T1-weighted MRI) scan. MRI data acquisition was performed using a standard Siemens Skyra 3 T scanner with a standard Siemens 32-channel RF receive head coil (voxel size = 1 mm^3^, field-of-view: 208 x 256 x 256 matrix with 5 min duration).

Raw T1-weighted MRI scans were processed to calculate grey matter volumes of subcortical regions at Imaging visit 1. FMRIB Software Library (FSL) was used to process the images. Detailed information on the pipeline implemented can be found at https://biobank.ctsu.ox.ac.uk/crystal/crystal/docs/brain_mri.pdf. In this study, we focused on the total grey matter volume of the amygdala (computed using left and right volumes: fields 25,888 and 25,889), as its association with neuroticism has been shown in previous studies^17,18,31,32^.

### Genetic data acquisition and preprocessing

Genotyping was conducted by Affymetrix using two custom-designed arrays. Approximately 50,000 participants were genotyped with the UK BiLEVE Axiom array, while the remaining ~450,000 participants were genotyped with the UK Biobank Axiom array^33^. The genetic data were converted from the initial.bgen file format to a PLINK-compatible binary format (.bed,.bim,.fam) using PLINK software (v2.0)^34^ and later underwent quality control (QC) procedures.

QC steps involved removing participants with discrepancies between genetically inferred and reported sex (fields 22001 and 31), sex chromosome aneuploidy (field 22019), and high missingness (field 22005). In addition, one subject for each pair of genetically related participants (field 22011) was excluded.

A variant-level QC was applied to the remaining dataset, retaining variants based on the following criteria: i) imputation score > 0.8, ii) minor allele frequency (MAF) > 0.1%, and iii) genotyping missingness < 2%. Variants with significant deviation from the Hardy-Weinberg equilibrium (*p*-value < 1e-6) were excluded.

To calculate genetic principal components (PCs), variants were first filtered to include only those with a Hardy-Weinberg equilibrium *p*-value > 1e-5, MAF ≥ 5%, and genotyping missingness ≤ 5%. Linkage disequilibrium (LD) pruning was then conducted using a window size of 1000 SNPs, a step size of 10 SNPs, and an r² threshold of 0.1. Principal component analysis (PCA) was performed on the LD-pruned dataset. Genetic analysis was restricted to individuals of European ancestry. The top 10 PCs were included in subsequent genetic association tests to account for population stratification. Participant filtering steps were performed using Python (v 3.7.3). Variant QC and PC generation were performed using PLINK software (v1.9)^34^.

### GWAS cohorts

This study conducted two separate genome-wide association studies (GWAS). The first analyzed binary phenotypes (pain-free vs. CMP), while the second focused on the neuroticism phenotype. The definitions of these phenotypes are provided in the previous sections. We restricted our analysis to participants of European ancestry and applied sample QC procedures (sex discrepancies, sex chromosome aneuploidy, high missingness, and removal of one subject for each pair of genetically related individuals), as outlined in the previous section. To construct the two GWAS cohorts (CMP and neuroticism GWAS), we first excluded all participants used in the CMP onset/recovery analyses to avoid overlap between the GWAS sample and the PRS target sample. For the neuroticism GWAS cohort, we further excluded individuals with missing values or uninterpretable responses (e.g., ‘Do not know’ or ‘Prefer not to answer’) in any of the twelve variables used to calculate the neuroticism score. The remaining participants were used to conduct the CMP and neuroticism GWAS. Variant-level QC was applied to both GWAS cohorts. Sex and genotyping batches were used as categorical covariates, while age and the top 10 PCs were included as quantitative covariates for both GWAS. The CMP and neuroticism GWASs were performed using the fast GLMM-based Genome-Wide Association tool (using the function *fastGWA-mlm-binary*)^35^ and the fast MLM-based Genome-Wide Association tool (using the function *fastGWA-mlm)*^36^, respectively.

### Polygenic risk scores

Two different PRSs were calculated for all the participants included in Datasets 1-3 and A-C for CMP onset and recovery analyses. PRS scores were derived using the CMP and neuroticism GWASs performed on the UK Biobank dataset, which included independent samples from the CMP onset and recovery cohorts. Using GWAS summary statistics, we estimated the posterior effect sizes of genetic variants using the PRS-CS tool^37^. PRS were then generated at the individual level using the *score* function in PLINK software (v1.9). This yielded i) CMP PRS and ii) neuroticism PRS for each participant included in the CMP onset/recovery datasets.

### Statistics and reproducibility

Logistic regression models were used to examine the impact of (i) neuroticism scores and (ii) neuroticism/CMP PRSs on the onset and recovery of CMP. Linear regression models were performed to assess the associations between (i) neuroticism scores and pain intensity phenotypes, (ii) neuroticism and CMP PRSs and pain intensity phenotypes, and (iii) neuroticism score and amygdala grey matter volume. Linear models were also used to assess group differences in the amygdala grey matter volumes.

To further elucidate the relationship between CMP PRS, neuroticism, and CMP onset/recovery, we conducted mediation analyses to examine whether neuroticism mediates the relationship between the CMP PRS and CMP onset/recovery. The independent variable in this model was the CMP PRS, the mediator was the neuroticism score, and the dependent variable was represented by the binary pain outcomes (CMP onset vs. no CMP onset, CMP recovery vs. persistence). Confidence intervals and p-values for the indirect effect were estimated using 1000 bootstrap iterations via the R *mediate* function.

The discovery cohorts (Datasets 1 and A) and two independent validation cohorts (Datasets 2 and 3; Datasets B and C) were used to ensure the reproducibility of the neuroticism score–CMP onset/recovery associations across independent samples. These associations were first tested in the discovery cohorts (Dataset 1 and A, TP1–TP3) and then independently validated in two separate cohorts (Dataset 2 and B, first validation; Dataset 3 and C, second validation). Analyses of neuroticism/CMP PRS – CMP onset/recovery associations, as well as mediation analyses, were performed in the discovery cohorts (Datasets 1 and A) and validated in the first validation cohorts (Datasets 2 and B). For analyses of neuroticism–pain intensity phenotype and neuroticism/CMP PRS–pain phenotype associations, only Datasets 2 and B were used, as no other datasets contained pain intensity information. The association between neuroticism score and amygdala grey matter volumes was examined using subsets of Dataset 3 and C with available imaging data. Statistical differences in neuroticism scores, PRS, and CMP PRS between datasets were tested using two-sided independent-sample t-tests to ensure the reliability of these measures.

Age, gender, BMI, smoking status, income, physical activity, Townsend deprivation index, education, and follow-up time were included as covariates (see Table S2 for additional details on the covariates used) in all linear and logistic regression analyses, as well as mediation analyses, due to their potential confounding effect in the association between chronic pain and neuroticism^38–44^. During PRS analysis, the top ten PCs were also included. When brain imaging data were added, total intracranial volume was inserted as an additional covariate.

False discovery rate (FDR) correction using the Benjamini-Hochberg method was used to account for multiple testing. Statistical significance was set at FDR-adjusted *p*  <  0.05. The logistic regression analyses were performed using the ‘*statsmodels’* package (v 0.14.2) in Python (v 3.7.3)^45^. Linear regression models and mediation analyses (using the *mediate* function available in the *mediation* package) were performed using the R programming language (v3.6.0)^46^.

We formulated our key hypotheses based on prior evidence linking neuroticism, CMP, and amygdala-mediated pain regulation, as outlined in the Introduction, and tested them using the UK Biobank dataset. No major analyses were added subsequently, except those conducted in response to reviewer comments. The study was not publicly preregistered.

## Results

### Characteristics of study participants

A total of 107,005 pain-free participants were identified at TP1. After applying exclusion procedures, Dataset 1 (Discovery cohort, TP1–TP3) consisted of a final number of 1830 participants who developed CMP and 7552 who remained pain-free at the follow-up (TP3). Among these participants, 7414 steady pain-free subjects and 1807 with CMP onset had genetic data available.

Similarly, in the CMP recovery analysis, 51,577 participants with CMP at TP1 were initially identified. The final cohort included in Dataset A (Discovery cohort, TP1–TP3) was comprised of 1863 participants with CMP at baseline (TP1) who later recovered and 1597 participants who remained with CMP at the follow-up assessment (TP3). Within this cohort, 1825 recovered participants and 1560 participants with persistent CMP had genetic data available.

The relatively high recovery rate ( > 50%) observed in Dataset A may partly reflect the extended follow-up period between TP1 and TP3 (mean ~10 years), which allowed sufficient time for symptom resolution. While differences in study design and follow-up duration prevent direct comparisons with other longitudinal studies, we do not believe this considerably influenced our results. The substantial participant numbers in both the recovered and persistent CMP groups are unlikely to have biased the analyses toward one class, which is preferable to having a strongly imbalanced class distribution.

Descriptive statistics for the discovery cohorts are reported in Table 1. The number of participants and demographic information for the different cohorts can be found in Tables S3–S5.

**Table 1:** Characteristics of study participants for CMP onset and recovery in Datasets 1 and A (TP1–TP3, Discovery Cohort)

Variable	Full sample	Steady pain-free	CMP onset
CMP Onset Data			
Number of Participants	9382	7552	1830
Age (years)	54.40 ± 7.44	54.38 ± 7.41	54.46 ± 7.57
Sex (N. Female)	4564 (48.64%)	3641 (48.21%)	923 (50.43%)
BMI (Kg/m^2^)	25.70 ± 3.64	25.56 ± 3.57	26.28 ± 3.88
Smoking			
Never	6055 (64.53%)	4972 (65.83%)	1083 (59.18%)
Previous	2846 (30.33%)	2211 (29,27%)	635 (34.69%)
Current	481 (5.12%)	369 (4.88%)	112 (6.12%)
Income			
Less than 18,000£	716 (7.63%)	554 (7.33%)	162 (8.85%)
18,000–30,999£	1759 (18.74%)	1386 (18.35%)	373 (20.38%)
31,000 –51,999£	2772 (29.54%)	2239 (29.64%)	533 (29.12%)
52,000–100,000£	3108 (33.12%)	2,525 (33.43%)	583 (31.85%)
Greater than 100,000£	1027 (10.94%)	848 (11.22%)	179 (9.78%)
Physical Activity (hours/week)	41.40 ± 38.70	40.96 ± 38.26	43.21 ± 40.54
Townsend Deprivation Index	−2.01 ± 2.63	−2.02 ± 2.63	−1.99 ± 2.60
Higher Education (college/university degree)	5005 (53.34%)	4105 (54.35%)	900 (49.18%)
Intermediate Education (A/AS levels or equivalent, O levels/GCSEs or equivalent)	2881 (30.70%)	2285 (30.25%)	596 (32.56%)
Low Education (none of the above)	1496 (15.94%)	1162 (15.38%)	334 (18.25%)
Neuroticism Score	2.88 ± 2.78	2.80 ± 2.74	3.22 ± 2.90
Follow-Up Time (years)	10.48 ± 2.68	10.46 ± 2.70	10.54 ± 2.61

CMP Recovery Data			
	Full Sample	Recovered from CMP	Persistent CMP
Number of Participants	3460	1863	1597
Age (years)	54.16 ± 7.47	54.39 ± 7.49	53.89 ± 7.44
Sex (N. Female)	1620 (46.82%)	858 (46.05%)	762 (47.71%)
BMI (Kg/m^2^)	26.46 ± 3.94	26.10 ± 3.70	26.88 ± 4.16
Smoking			
Never	2060 (59.53%)	1109 (59.52%)	951 (59.54%)
Previous	1207 (34.88%)	644 (34.56%)	563 (35.25%)
Current	193 (5.57%)	110 (5.90%)	83 (5.19%)
Income			
Less than 18,000£	305 (8.81%)	160 (8.58%)	145 (9.07%)
18,000 to 30,999£	660 (19.07%)	360 (19.32%)	300 (18.78%)
31,000 to 51,999£	1046 (30.23%)	561 (30.11%)	485 (30.36%)
52,000 to 100,000£	1125 (32.51%)	602 (32.31%)	523 (32.74%)
Greater than 100,000£	324 (9.36%)	180 (9.66%)	144 (9.01%)
Physical Activity (hours/week)	42.92 ± 40.54	41.37 ± 39.48	44.74 ± 41.68
Townsend Deprivation Index	−2.08 ± 2.62	−2.11 ± 2.61	−2.03 ± 2.62
Higher Education (college/university degree)	1597 (46.15%)	887 (47.61%)	710 (44.45%)
Intermediate Education (A/AS levels or equivalent, O levels/GCSEs or equivalent)	1166 (33.69%)	639 (34.29%)	527 (32.99%)
Low Education (none of the above)	697 (20.14%)	337 (18.08%)	360 (22.54%)
Neuroticism Score	3.68 ± 3.01	3.52 ± 2.98	3.86 ± 3.04
Follow-Up Time (years)	10.44 ± 2.66	10.43 ± 2.62	10.45 ± 2.70

*CMP* Chronic Musculoskeletal Pain, *TP* Timepoint. The Townsend Deprivation Index is a measure of socioeconomic deprivation.

Neither neuroticism PRS nor the CMP PRS differed between Datasets 1-2 (neuroticism PRS: *p* = 0.401, CMP PRS: *p* = 0.305) or between Datasets A-B (neuroticism PRS: *p* = 0.766, CMP PRS: *p* = 0.302). For neuroticism scores, no significant differences were observed between Datasets 1-2-3 (*p* = 0.580; A vs. C: *p* = 0.088; B vs. C: *p* = 0.071) and Datasets A-B-C (A vs. B: *p* = 0.785; A vs. C: *p* = 0.212; B vs. C: *p* = 0.169).

The CMP GWAS included 150,739 pain-free and 120,581 CMP individuals, and the neuroticism GWAS included 329,368 samples. Flowcharts detailing the dataset construction process for these GWAS cohorts can be found in Fig. S5.

### Associations between neuroticism score and CMP onset/recovery

Logistic regression analyses showed that higher neuroticism scores, assessed at TP1 in a cohort of pain-free individuals, were significantly associated with an increased risk of CMP onset at TP3, using data from the discovery cohort (Dataset 1: TP1–TP3; *p* < 0.001, FDR < 0.05, OR = 1.055, 95% CI: 1.036–1.074). These findings were replicated in the other validation cohorts (Table 2, Table S6).

**Table 2:** Logistic regression results for the association between neuroticism score and CMP onset and recovery

Dataset	*p*-value (uncorrected)	p FDR	Odds Ratio [95% CI]
CMP Onset Data			
Dataset 1 (TP1–TP3)	7.92 e-9	3.16 e-8	1.055 [1.036–1.074]
Dataset 2 (TP1–PQ)	1.82 e-13	1.45 e-12	1.042 [1.031–1.054]
Dataset 3 (TP3–TP4)	1.08 e-4	2.66 e-4	1.126 [1.058–1.199]
CMP Recovery Data			
Dataset A (TP1–TP3)	0.001	0.002	1.038 [1.014–1.062]
Dataset B (TP1–PQ)	0.002	0.0032	1.025 [1.008–1.041]
Dataset C (TP3–TP4)	0.949	0.949	0.997 [0.924–1.075]

*TP* Timepoint, *PQ* Pain Questionnaire, *CMP* Chronic Musculoskeletal Pain. All the models were adjusted for age, gender, BMI, income, smoking, physical activity, Townsend deprivation index, education, and follow-up time. The p-values, both uncorrected and after FDR correction, and odds ratios associated with neuroticism score are reported. All logistic regression coefficients were tested using two-sided tests.

Similarly, we evaluated the impact of neuroticism on recovery from CMP by computing neuroticism scores at TP1 in a cohort of CMP individuals. Results of the logistic regression analyses showed that higher neuroticism scores were significantly associated with increased risk of persistent CMP at TP3 in the discovery cohort (Dataset A: TP1–TP3; *p* = 0.001, FDR < 0.05, OR = 1.038, 95% CI: 1.014–1.062). These significant associations were replicated in the first validation cohort (Dataset B: *p* = 0.002) but were not significant in the second validation cohort (Dataset C: *p* = 0.949). Results for all cohorts can be found in Table 2 and Table S7.

### Associations between neuroticism/CMP PRSs and CMP onset/recovery

We then evaluated the influence of the CMP and neuroticism PRSs on both CMP onset and recovery using subsets of individuals with available genetic data. To maximize statistical power, we focused on the two datasets with the largest sample sizes: Dataset 1 and A (TP1 to TP3) and Dataset 2 and B (TP1 to PQ).

Higher CMP PRS in pain-free individuals was associated with increased risk of CMP onset in the discovery cohort (Dataset 1: TP1–TP3; *p* < 0.001, FDR < 0.05, OR = 1.197, 95% CI: 1.135–1.262). These findings were replicated in the first validation cohort Dataset 2 (TP1–PQ; *p* < 0.001, FDR < 0.05, OR = 1.137, 95% CI: 1.101–1.173) (Table 3, Table S8). Furthermore, higher neuroticism PRS was significantly associated with increased risk of CMP onset in both the discovery cohort (Dataset 1: TP1–TP3; *p* < 0.001, FDR < 0.05, OR = 1.093 95% CI: 1.038–1.152) and in Dataset 2 (TP1–PQ; *p* = 0.027, FDR < 0.05, OR = 1.035, 95% CI: 1.003 – 1.068). (Table 3, Table S9).

**Table 3:** Logistic regression results for the associations between CMP polygenic risk score (PRS)/neuroticism PRS and CMP onset/recovery

Dataset	PRS	p-value	p FDR	Odds Ratio [95% CI]
CMP Onset Data				
Dataset 1 (TP1 – TP3)	CMP PRS	3.76 e-11	1.50 e-10	1.197 [1.135–1.262]
	Neuroticism PRS	7.66 e-4	0.0015	1.093 [1.038–1.152]
Dataset 2 (TP1 – PQ)	CMP PRS	2.55 e-15	2.04 e-14	1.137 [1.101–1.173]
	Neuroticism PRS	0.027	0.031	1.035 [1.003–1.068]

CMP Recovery Data				
Dataset A (TP1 – TP3)	CMP PRS	4.78 e-6	1.27 e-5	1.178 [1.098–1.264]
	Neuroticism PRS	0.023	0.030	1.082 [1.010–1.160]
Dataset B (TP1 – PQ)	CMP PRS	0.010	0.016	1.064 [1.014–1.117]
	Neuroticism PRS	0.083	0.083	1.042 [0.994 – 1.093]

*ATP* Timepoint, *PQ* Pain Questionnaire, *CMP* Chronic Musculoskeletal Pain. All the models were adjusted for age, gender, BMI, income, smoking, physical activity, townsend deprivation index, education, follow-up time, and the first 10 Principal Components. The *p*-values, both uncorrected and after False Discovery Rate (FDR) correction, and odds ratios are reported. All logistic regression coefficients were tested using two-sided tests.

In the CMP PRS analysis of individuals with CMP, a significant association between the CMP PRS and increased risk of persistent CMP was found in Dataset A (TP1–TP3; *p* < 0.001, FDR < 0.05, OR = 1.178, 95 % CI: 1.098–1.264). These results were replicated in Dataset B (TP1–PQ; *p* = 0.01, FDR < 0.05, OR = 1.064, 95% CI: 1.014–1.117) (Table 3, Table S8). Moreover, higher neuroticism PRS was significantly associated with increased risk of persistent CMP in the discovery cohort (Dataset A: TP1–TP3; *p* = 0.023, FDR < 0.05, OR = 1.082, 95% CI: 1.010 – 1.160). However, this association showed no significance in Dataset B, the primary validation cohort (TP1–PQ; *p* = 0.083, FDR = 0.083, OR = 1.042, 95% CI: 0.994–1.093). Due to the absence of replication, these findings should be interpreted with caution (Table 3, Table S9).

### Mediation analysis results

**Fig. 2:**
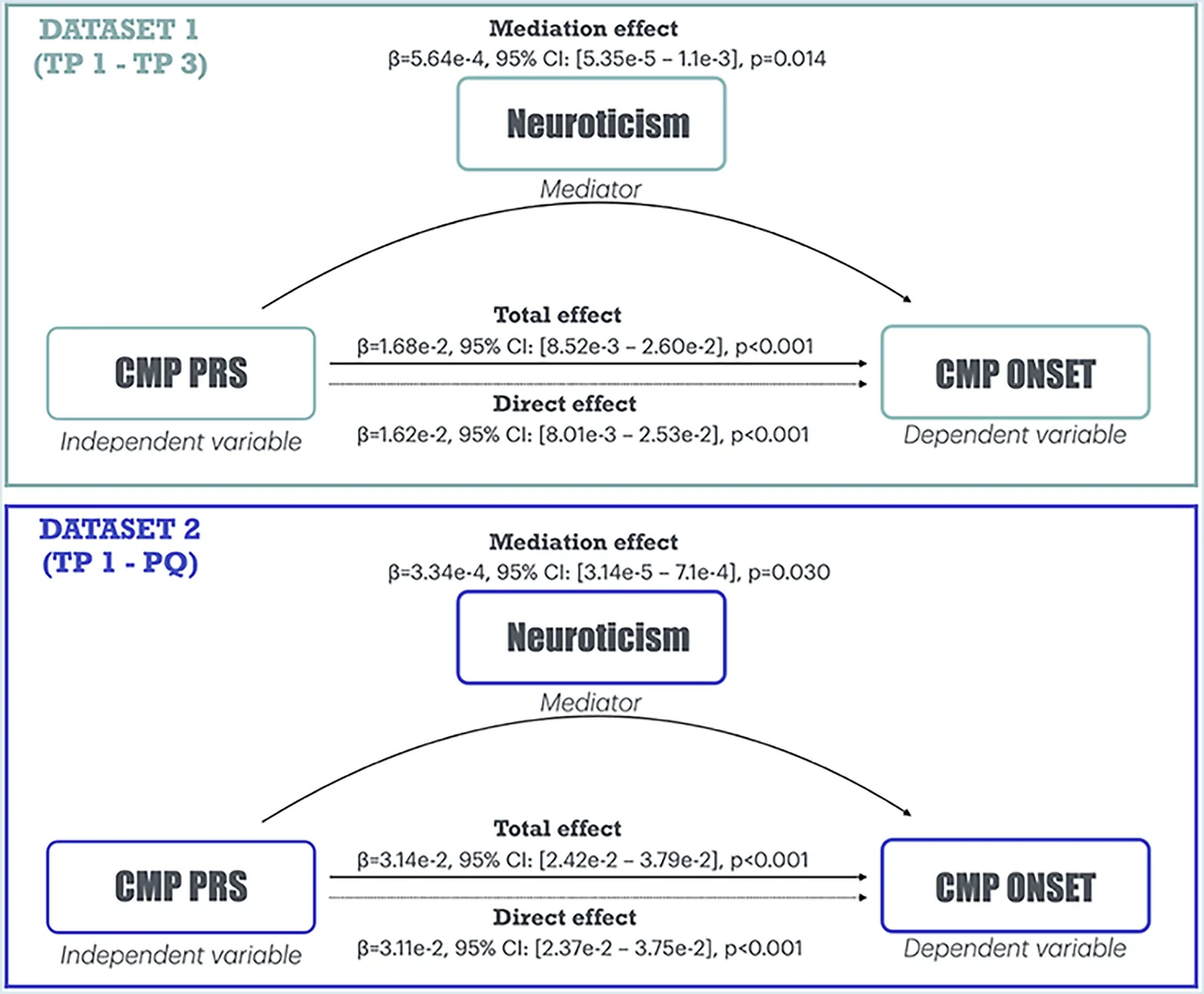
Results of the mediation analysis using the chronic musculoskeletal pain (CMP) onset Datasets 1 (top panel) and 2 (bottom panel). Neuroticism served as the mediator of the association between the CMP polygenic risk score (PRS; independent variable) and binary pain outcome (steady pain-free vs. CMP onset). Covariates included age, gender, BMI, smoking status, income, physical activity, Townsend deprivation index, education, follow-up time, and the top ten Principal Components (PCs). Significance in the discovery cohort (Dataset 1) was adjusted for multiple comparisons using a Bonferroni correction for two tests (*p* < 0.05/2 = 0.025). The mediation analyses were conducted using two-sided tests.

Since we observed significant associations between neuroticism score and CMP onset/recovery at both the phenotypic and genetic levels, we conducted mediation analyses to further elucidate the interplay between these variables. Specifically, in the first mediation analysis conducted on the discovery cohort, we tested whether neuroticism mediated the association between the CMP PRS and CMP onset/recovery. Results showed that neuroticism significantly mediated the relationship between the CMP PRS (independent variable) and subsequent CMP onset (dependent binary variable: CMP onset vs. no CMP onset) after p-value correction in Dataset 1 (p_uncorrected_ = 0.014, p_corrected_ < 0.05/2 = 0.025, Bonferroni correction for onset and recovery analyses). These results were replicated in Dataset 2 (Table 4 and Fig. 2).

**Table 4:** Mediation analysis results

Independent Variable	Mediator	Dependent Variable	Mediation Effect	Direct Effect	Total Effect
**CMP Onset Dataset 1 (TP1 – TP3)**					
CMP-PRS	Neuroticism	CMP Onset/No Onset	*β* = 5.64e-4 [5.35e-5 – 1.1e-3], *p* = 0.014	β = 1.62e-2 [8.01e-3 – 2.53e-2], *p* < 0.001	β = 1.68e-2 [8.52e-3 – 2.60 e-2], *p* < 0.001
**CMP Onset Dataset 2 (TP1 – PQ)**					
CMP-PRS	Neuroticism	CMP Onset / No Onset	*β* = 3.34e-4 [3.14e-5 – 7.1e-4], *p* = 0.030	*β* = 3.11e-2 [2.37e-2 – 3.75e-2], *p* < 0.001	*β* = 3.14e-2 [2.42e-2 – 3.79e-2], *p* < 0.001
**CMP Recovery Dataset A (TP1 – TP3)**					
CMP-PRS	Neuroticism	Recovery / Persistence	*β* = 8.59e-4 [6.28e-5 – 2.1e-3], *p* = 0.032	β = 4.02e-2 [2.29e-2 – 5.61e-2], p < 0.001	β = 4.12e-2 [2.39e-2 – 5.70e-2], *p* < 0.001
**CMP Recovery Dataset B (TP1 – PQ)**					
CMP-PRS	Neuroticism	Recovery / Persistence	*β* = 8.63e-5 [−3.20e-4 – 0.0005], *p* = 0.536	1.56e-2 [3.94e-3 – 2.71e-2], *p* = 0.012	1.56e-2 [4.02e-3 – 2.73e-2], *p* = 0.012

*PRS* Polygenic Risk Score, *CMP* Chronic Musculoskeletal Pain, *TP* Timepoint, *PQ* Pain Questionnaire. All the models were adjusted for age, gender, BMI, smoking, income, townsend deprivation index, education, physical activity, follow-up time, and the first 10 Principal Components (PCs). All the continuous variables were z-score normalized. All the coefficients were tested using two-sided tests.

No significant results were found after p-value correction when applying the same mediation analyses on the CMP recovery cohort (Datasets A-B), using neuroticism as mediator, the CMP PRS as the independent variable, and the binary pain outcome (CMP recovery vs. persistence) as the dependent variable. Results of the mediation analysis are shown in Table 4 and Figure S6.

### Impact of neuroticism score and neuroticism/CMP PRSs on pain characteristics

Furthermore, we used baseline variables measured at TP1—including neuroticism score, as well as neuroticism and CMP PRSs—to examine their impacts on pain phenotypes assessed at the follow-up timepoint (PQ). Datasets 2 and B were the only datasets with pain intensity information available.

Using the CMP onset data, neuroticism score showed a significant positive association with the total pain intensity score (FDR < 0.05), worst pain intensity score (FDR < 0.05), and the number of CMP sites (FDR < 0.05). Additionally, the neuroticism PRS showed a significant positive association with these phenotypes (all FDR < 0.05). No associations were detected between the CMP PRS and pain intensity phenotypes (Table 5).

**Table 5:** Linear regression results for the associations between neuroticism score/neuroticism and CMP polygenic risk score (PRS) and pain characteristics for both CMP onset and recovery analyses

Independent Variable	Pain Outcome	*N*	*p*-value	p FDR	*β* ± SE
**CMP Onset Data**					
Neuroticism Score	Total Pain Intensity Score	18,740	1.55 e-14	2.79 e-13	0.054 ± 0.007
	Worst Pain Intensity		4.08 e-14	3.21 e-13	0.033 ± 0.004
	Number of CMP sites		5.36 e-14	3.21 e-13	0.014 ± 0.001
Neuroticism PRS	Total Pain Intensity Score	18,407	0.007	0.012	0.052 ± 0.019
	Worst Pain Intensity		0.002	0.0045	0.037 ± 0.012
	Number of CMP sites		0.008	0.013	0.014 ± 0.005
CMP PRS	Total Pain Intensity Score	18,407	0.425	0.425	0.048 ± 0.061
	Worst Pain Intensity		0.221	0.248	0.049 ± 0.040
	Number of CMP sites		0.213	0.248	0.021 ± 0.017
**CMP Recovery Data**					
Neuroticism Score	Total Pain Intensity Score	7217	9.77 e-5	3.51 e-4	0.071 ± 0.018
	Worst Pain Intensity		0.0002	6.00 e-4	0.036 ± 0.009
	Number of CMP sites		3.80 e-6	1.71 e-5	0.020 ± 0.004
Neuroticism PRS	Total Pain Intensity Score	7104	0.006	0.012	0.146 ± 0.053
	Worst Pain Intensity		0.002	0.0045	0.083 ± 0.029
	Number of CMP sites		0.031	0.046	0.028 ± 0.012
CMP PRS	Total Pain Intensity Score	7104	0.112	0.155	0.241 ± 0.151
	Worst Pain Intensity		0.265	0.280	0.089 ± 0.080
	Number of CMP sites		0.147	0.189	0.054 ± 0.037

*CMP* Chronic Musculoskeletal Pain, *PRS* Polygenic Risk Score, *FDR* False Discovery Rate. The *p*-values, both uncorrected and after False Discovery Rate (FDR) correction, and odds ratios are reported. All linear regression coefficients were tested using two-sided tests.

In the CMP recovery cohort, a higher neuroticism score was associated with higher total pain intensity (FDR < 0.05), higher worst pain intensity (FDR < 0.05), and a greater number of CMP sites (FDR < 0.05). Neuroticism PRS also showed a significant positive association with pain intensity phenotypes (all FDR < 0.05). Conversely, CMP PRS showed no significant associations with pain intensity phenotypes (Table 5).

### Association between amygdala grey matter volume and neuroticism score

Using Dataset 3 (TP3–TP4), we first evaluated group differences (steady pain-free vs. CMP onset/CMP recovery vs. persistent CMP) at TP3. A significant difference in amygdala grey matter volume was detected between steady pain-free and CMP onset groups (two-sided independent-samples t-test: p = 0.0037, mean ± SD group steady pain-free: 4019.86 ± 466.1, mean ± SD group CMP onset: 4112.38 ± 466.2). Participants with CMP onset showed larger amygdala grey matter volumes compared to those who remained pain-free across assessments. In addition, we found that neuroticism scores had a significant positive association with the amygdala grey matter volumes (*p* = 0.014, β = 10.66 ± 4.37).

In the CMP recovery analysis using Dataset C (CMP recovery vs. persistent CMP), no differences were found in the amygdala grey matter volumes (two-sided independent-samples t-test: *p* = 0.693). Moreover, no significant association was found between amygdala grey matter volumes and neuroticism score (*p* = 0.894, *β* = 0.82 ± 6.2).

## Discussion

Our analysis of the UK Biobank dataset across multiple timepoints revealed that neuroticism influences CMP onset, as well as recovery and pain characteristics, with both neuroticism and genetic risk playing a significant role in these outcomes. Key findings include: (1) higher neuroticism scores were significantly associated with an increased risk of CMP onset and persistence, greater pain intensities, and a broader distribution of pain across multiple sites; (2) CMP and neuroticism polygenic risk scores (PRSs) were significantly associated with increased risk of CMP onset and persistence; (3) neuroticism significantly mediated the association between CMP PRS and CMP onset, although the effect size was modest; (4) no significant mediation effect was observed for CMP recovery after p-value correction; (5) neuroticism PRS was significantly associated with pain intensity phenotypes in both CMP onset and recovery cohorts, whereas CMP PRS showed no associations with pain intensity; and (6) increased amygdala volume was observed in participants who later developed CMP, with significant correlations with neuroticism in the CMP onset cohort but not in the recovery group. These findings were validated across different validation cohorts.

By following pain-free individuals over time to track the onset of CMP, we found that higher neuroticism scores are associated with increased risk of CMP onset. The findings were replicated across multiple cohorts. In a separate analysis, we found that neuroticism significantly influenced recovery outcomes from CMP, with higher neuroticism associated with an increased risk of persistent CMP.

Literature suggests that subjects with chronic pain are more vulnerable to experience negative emotional states^47,48^. Reciprocally, neurotic behaviors affect pain perception, exacerbating pain catastrophizing^6,49^. Moreover, negative mood in the self-ability to deal with pain intensifies pain sensation, lowering pain tolerance and impacting pain outcomes^50,51^. Psychological interventions have been designed for chronic pain treatment, underscoring the role of negative emotional states in the persistence of pain over time^52,53^. However, their cross-sectional designs limit the understanding of the causal relationship between the two conditions.

Consistent with previous research, our findings underscore an interplay between psychological states, such as neuroticism and CMP conditions. Importantly, our study provides evidence of a longitudinal link between negative emotional states and the risk of developing CMP in a cohort of pain-free individuals, identifying neuroticism as a significant risk factor for CMP onset. In addition, individuals with higher neuroticism are more likely to report persistent CMP, supporting the idea that psychological resilience and lower emotional distress contribute to better pain management, leading to improved recovery outcomes.

Our findings highlight the crucial impact of neuroticism on pain outcomes for both CMP onset and recovery, emphasizing the importance of emotional health in shaping pain conditions and offering insights into potential strategies for chronic pain prevention and management.

At the genetic level, our findings indicate that a higher PRS for CMP is significantly associated with CMP outcomes, including both onset and recovery, though it was not significantly associated with pain intensity phenotypes, indicating that the genetic factors underlying chronic pain susceptibility may differ from those influencing pain severity. A higher neuroticism PRS was also linked to CMP trajectories, as well as increased pain intensities and sites. However, while this association demonstrated a significant positive correlation in the discovery cohort (Dataset A; *p* = 0.023), it did not reach statistical significance in the primary validation cohort (Dataset B; *p* = 0.083). Due to lack of replication, these findings should be interpreted cautiously.

At the phenotypic level, neuroticism has been associated with heightened pain perception and increased pain-related anxiety, suggesting that negative mental health states may exacerbate pain experiences by influencing pain sensitivity, emotional regulation, and stress responses^5,49,54^.

Furthermore, the bidirectional relationship between negative mental health states, such as neuroticism, and chronic pain conditions is supported by biological evidence, with studies establishing a causal genetic link between chronic pain and neuropsychiatric disorders, confirming the interplay between mental health and pain conditions^55^.

Our results suggest that genetic predisposition to neuroticism may influence pain-related biological mechanisms, potentially affecting pain modulation, persistence, and recovery. This supports and expands upon previous evidence of the comorbidity and shared genetic architecture between neuroticism and chronic pain conditions. The longitudinal design of our study further strengthens the understanding of their causal relationship, suggesting that biological factors linked to neuroticism contribute to both the onset and persistence of CMP. Moreover, consistent with the concept that pain catastrophizing is associated with negative mental states, our results demonstrate that genetic risk for neuroticism also plays a role in increased pain vulnerability.

Our mediation analysis reveal that neuroticism mediates the relationship between CMP polygenic risk scores (PRS) and CMP onset, suggesting a potential psychological mechanism through which genetic predisposition may contribute to chronic pain outcomes. However, the observed effect sizes were small and should not be overinterpreted in terms of clinical impact. The indirect effects attributable to neuroticism (Dataset 1: *β* = 5.64 × 10^−4^; Dataset 2: *β* = 3.34 × 10^−4^) were substantially smaller than their corresponding total effects (Dataset 1: *β* = 1.68 × 10^−2^; Dataset 2: *β* = 3.14 × 10^−2^), indicating that neuroticism accounts for only a minor proportion of the overall genetic association with CMP onset. This pattern is further reflected in the logistic regression models, where odds ratios for neuroticism score (OR range: 1.025–1.126; Table 2) and neuroticism PRS (OR range: 1.035–1.093; Table 3) were modest and must be contextualized against baseline pain incidence rather than interpreted as deterministic predictors. Given a baseline CMP incidence of approximately 19.5% in our onset cohort (1830 of 9382 pain-free participants; Table 1), an OR of 1.05 translates to an absolute risk increase of roughly one percentage point per neuroticism unit, small at the individual level but potentially meaningful when aggregated across large populations.

These modest effect sizes are nonetheless consistent with prior literature^6,49,56^, which has similarly reported small but significant associations between neuroticism and pain perception, supporting its role as a psychological vulnerability factor influencing pain. Direct comparisons across studies remain limited by differences in outcome definitions and reporting metrics. Small effects are also not unexpected given that both neuroticism and chronic pain are highly polygenic traits, with PRS typically explaining only a modest proportion of phenotypic variance.

A notable distinction emerged between onset and recovery outcomes. While neuroticism significantly mediated the pathway from genetic risk to CMP onset, no significant mediation effect was observed for recovery. This asymmetry suggests that neuroticism primarily acts as a vulnerability factor amplifying genetic susceptibility in pain-free individuals, whereas recovery is likely governed by a distinct and more complex interplay of biological, psychological, and environmental factors. These diverse influences also help explain the considerable individual variability in pain trajectories more broadly. Neuroticism should therefore be understood as one component within a broader multifactorial risk profile. Our findings do not constitute standalone clinical predictors but rather reflect a complex framework in which many factors contribute to CMP onset and recovery. Further research is needed to identify additional mediating pathways and clarify the mechanisms linking genetic risk to CMP outcomes across different stages of pain trajectory.

During the first imaging assessment (TP3), we observed a bilateral increase in amygdala grey matter volume among healthy participants who subsequently developed CMP by TP4, compared to those who remained pain-free throughout the study period. Within this group, the increase in amygdala grey matter volume was positively correlated with neuroticism scores, suggesting that structural amygdala changes in future CMP developers may be partly related to the heightened emotional sensitivity characteristic of high-neuroticism individuals. Notably, this association was not detected in participants already experiencing CMP at the time of imaging, nor were differences in amygdala grey matter volume observed in the recovery analysis (Dataset C; CMP recovery vs. persistent CMP).

The presence of amygdala enlargement specifically in individuals who later developed CMP, but not in those already living with chronic pain, suggests that increased amygdala volume may represent a neural risk marker for CMP onset rather than solely a structural consequence of established chronic pain. This interpretation is further supported by the observed association between amygdala volume and neuroticism. These findings are consistent with previous studies reporting increased amygdala volume in chronic pain populations^19,57^. We extend this literature by suggesting that amygdala enlargement may precede the development of CMP. However, an alternative interpretation is that individuals in the incident CMP group may have already experienced subthreshold or unreported pain at baseline, which could have contributed to amygdala-related changes. The absence of significant amygdala volume differences in Dataset C, in which all participants had established chronic pain at baseline, may be explained by the fact that pain-related structural changes had already occurred in both groups before imaging, leaving no detectable difference between them. Further longitudinal and mechanistic studies are needed to validate these findings and clarify whether amygdala enlargement represents a pre-existing vulnerability factor, an early marker of subclinical pain, or both.

Functional MRI studies have established an association between neuroticism and the amygdala, showing that neurotic behaviors trigger the activation of the amygdala as part of a physiological response to distress^18^. Moreover, studies focused on structural changes in the brain have shown a correlation between high neuroticism and increased grey matter concentration in the amygdala. A recent meta-analysis highlighted the positive correlation between neuroticism and the amygdala grey matter volume^17^. In another study, this association was consistently observed only in healthy controls, but not in individuals with depression^31^. Additionally, an increase in amygdala grey matter volume was also observed in healthy individuals with trait anxiety, as well as in healthy first-degree relatives of patients with major depressive disorder^58,59^.

Aligning with previous research, our findings demonstrate the impact of neuroticism on increased grey matter volume in the amygdala. Our results suggest negative personality traits and amygdala grey matter volume as physiological processes that may precede the onset of CMP, demonstrating their potential as predictors for CMP development.

### Limitations

This study has several limitations that should be acknowledged. The variables used for the analysis, such as neuroticism and incidence of chronic pain, were self-reported and may introduce biases. Moreover, the lack of information on previous pain history (prior to UKB enrollment) limits the ability to account for potential confounding factors. The possibility of reverse causation should also be considered, as prior pain history could influence both neuroticism and future pain outcomes. Nonetheless, self-reported measures are standard in large-scale epidemiological cohort studies. The longitudinal design of the onset cohort provides some protection against reverse causation, as neuroticism is measured in pain-free individuals across the onset datasets (Datasets 1, 2, and 3), allowing for the detection of neuroticism before the development of CMP. However, participants could have had prior unreported pain that influenced their baseline neuroticism. In the recovery cohort, where participants already had CMP at baseline, reverse causation remains a substantial concern.

Our study excluded participants with anxiety, depression, long-term disability, and related conditions to isolate neuroticism-specific effects and reduce confounding factors. However, we acknowledge that these are prevalent comorbidities within populations experiencing chronic pain, and their exclusion may have constrained the clinical generalizability of our findings to the broader chronic pain population. Additionally, our exclusion criteria could introduce selection bias toward milder clinical presentations of chronic pain, as we omitted individuals with long-term disability, which can be a manifestation of extreme, disabling pain. This can result in individuals with more severe pain being underrepresented. Moreover, while our exclusion criteria using the ICD-9/10 codes accounted for a wide range of inflammatory and autoimmune musculoskeletal conditions, we acknowledge that residual heterogeneity may remain and could influence our results. Despite these limitations, these exclusions were necessary to isolate the specific effects of neuroticism from those of confounds.

Additionally, our strategy to prioritize the discovery cohort when assigning participants could have reduced the representation of the validation cohorts, which may introduce selection bias and potentially affect generalizability. Participants also were not assessed systematically throughout the follow-up period, making it impossible to determine whether their neuroticism or chronic pain states changed between the baseline and follow-up assessments. While our study used multiple timepoints across different datasets, each cohort in our study used two assessment timepoints (a baseline and a single follow-up), so within-person pain trajectories between each timepoint could not be characterized. Despite adjustment for multiple covariates (Table S2), unmeasured variables such as early life adversity, sleep, and socioeconomic stress over the longitudinal study could have contributed to pain outcomes. Thus, causal inference remains limited. Finally, the genetic analysis was restricted to European ancestry, which limits the generalizability of the PRS to non-European populations. Future research should focus on replication in ancestrally diverse cohorts.

### Conclusion

In this study, we emphasize the influence of neuroticism and genetic risk on the onset and recovery of chronic musculoskeletal pain, along with its influence on amygdala grey matter volume. Additionally, we demonstrated how psychological factors can interplay with the associations between genetic risk and amplified pain outcomes. We found associations between genetic risk for neuroticism and CMP onset; however, its role in CMP recovery was less clear. Our findings suggest that negative emotional states, like neuroticism, along with genetic factors, could serve as potential biomarkers for CMP prevention and management. Nevertheless, the small effect sizes observed throughout reinforce that these factors operate as contributing elements within a broader multifactorial framework, rather than as deterministic predictors of pain outcomes.

## Supplementary information

Transparent Peer Review file

Supplemental Information

## Data Availability

The data used in this study is publicly available (additional details can be found at https://biobank.ndph.ox.ac.uk/showcase/). Source data underlying the figures presented in the main manuscript are provided in Suppl. Data 1. All relevant data generated in this study are available from the corresponding author upon reasonable request.
