# Supplementary material for: The roles of neuroticism, genetic susceptibility, and amygdala structure in chronic musculoskeletal pain onset and recovery
Source: Commun Med (Lond). 2026 Jul 6;6:567. doi: 10.1038/s43856-026-01754-9 (PMC13719999; doi:10.1038/s43856-026-01754-9)
Supplement: Supplementary file 1 [file 43856_2026_1754_MOESM1_ESM.pdf]

nature portfolio

Peer Review File

The roles of neuroticism, genetic susceptibility, and amygdala structure in chronic musculoskeletal pain onset and recovery

Corresponding Author: Professor Jian Kong

This file contains all reviewer reports in order by version, followed by all author rebuttals in order by version.

Version 0:

Reviewer comments:

Reviewer #1

(Remarks to the Author)
This study examined the interaction between neuroticism (both behavioral and PRS), amygdala grey matter volume, and pain (CMP, behavioral and PRS) using the UK Biobank data over different time points (2006-2019). Participants were divided into the CMP and the Control (No-Pain) groups. Pain onset, intensity, and recovery were also considered as components of pain. Both neuroticism and CMP PRSs were linked to higher CMP and a lower chance of recovery. Both behavioral and PRS of neuroticism were associated with pain intensity. The CMP onset group at a later time point showed increased amygdala volumes, compared to the group without pain onset. The authors reported neuroticism as a mediator in the relationship between CMP PRS and CMP onset and concluded the role of neuropsychological effects of neuroticism on the 'causal' relationship between genetic risk of pain and actual onset of pain. Logistic and linear regressions were used to show the relationships reported in the study.

This is a very well-written manuscript. Overall structure and organization of study findings are excellent. UK Biobank datasets are a well-established and tested dataset. The examination of behavioral and genetic factors in pain longitudinally seems to be a major contribution of the study.
However, I have a reservation about the novelty of the study findings. Previous studies have noted the relationship between neuroticism and pain, although it is difficult to find the directionality of the relationship. This study reports those relationships over different time points. The main 'novel' finding seems to be the mediating role of neuroticism on the relationship between CMP PRS and CMP onset. To me, the results may not be sufficient to make such conclusion statistically, in addition to the null findings from the recovery cohort. The result of uncorrected $p < .03$ does not seem to reflect substantial evidence for the role of the mediator variable.

In Figure 2, the direct effect of the relationship between CMP PRS and onset almost explains the total effect of the relationship between them, while the effects are relatively small, assuming betas are standardized effect sizes. The size of the mediation effect is even tinier. Considering the sample sizes of the study, it is not unreasonable not to exclude the possibility of a false positive. Separate associations among variables are also reported. However, those associations do not necessarily make convincing evidence for the mediating effects of neuroticism on pain, as claimed in the discussion and the abstract. More substantial data to back up the mediation effect of neuroticism could be helpful to convince readers about the novel findings of this study.

Reviewer #2

(Remarks to the Author)
This study explores the role of neuroticism, genetic risk, and amygdala structure in the development and recovery of chronic musculoskeletal pain (CMP) using longitudinal data from the UK Biobank of those who did and did not have CMP at baseline as well as those who did and did not recover at follow-up. The authors report significant evidence for genetic and causal relationships between neuroticism and CMP onset and recovery, supporting a neurobiological mechanism linking psychological states to chronic pain vulnerability. This is an impressive manuscript with important implications for the field. However, I have a few concerns:

ABSTRACT:
1) Overall, I think the abstract succinctly sums up the results and importance of the paper. I would encourage the authors to include some of the null findings as well (neuroticism did not mediate CMP PRS-CMP recovery; CMP PRS was not associated with pain intensity phenotypes, amygdala volume related to neuroticism in CMP onset participants but not CMP recovery), as this could be of interest to individuals skimming the abstract.

INTRODUCTION:
2) I think there needs to be a more thorough discussion of the background of the overlapping biological mechanisms. As it stands, they are convincing but too vague – for example, are there specific genes that are related to both neuroticism and CMP? Have people theorized as to what these genetic mechanisms mean for both neuroticism and CMP? What about the purported structural and functional changes you mention – you say that these are associated with both neuroticism and CMP but don't discuss the direction (greater connectivity, less volume, etc)? I would like to see more concrete evidence to support what you are saying.

3) You seem to suggest that there is already a plethora of work linking neuroticism and pain perception and persistence, but only cite one paper. I'd like to see more work cited/discussed to support this tone.

4) This may have more to do with the methods, but what is the justification for only using CMP participants? Why disregard other types of chronic pain? Do you think there is something unique about the relationship between neuroticism and CMP vs. neuroticism and chronic pain in general? If so, a much more detailed explanation is required in the introduction.

METHODS:
5) Overall, I felt the methods were difficult to follow given the complexity of the sample used. It was not until I examined the flowcharts in the supplementary that I full understood how each dataset was created (regarding which participants were selected for which dataset and why). I think it would be incredibly beneficial if you included a timeline figure to include each visit, the dates, which variable that you are using were collected at each visit, etc. This could be included in Figure 1 as Part A or B or stand alone as its own figure. Delineating which study visits were used for which cohorts and the final N for each would be beneficial here.

6) You should report the exclusion criteria in the main body of the paper and not the supplementary materials.

7) What is the justification for using the study visits selected? I know that PQ was selected because it was the only visit that reported pain intensities (this is noted under the pain intensity and pain sites phenotypes section of the methods but should be made clearer much earlier). But why not use TP2 at all? I think there is a need for a stronger discussion as to why you used each visit for each of the datasets.

8) Relatedly, what if a participant reported pain starting at TP2, pain free again by TP3 but developed pain again by PQ? How would you classify that participant? Such instances could be made clearer.

9) Although I appreciate wanting to maintain the consistency for Datasets 1-3, I found it a bit confusing given that it is technically 6 different datasets that are separated by whether they are looking at onset or recovery as the main outcome of interest. Perhaps it might be worthwhile to rename it as Datasets 1-3 and corresponding Datasets A-C? It maintains the similarity that you initially wanted and makes it clearer that these are totally different datasets. Just a suggestion.

10) Did you consider that some individuals that reported having pain "all over" could have fibromyalgia, a condition of widespread musculoskeletal pain? What is the advantage of excluding these participants?

11) Is the neuroticism survey a validated survey?

12) I wonder what is the purpose of calculating a sum of pain intensities across each pain site. Is this truly the best approach to capture somebody having "more" pain?

13) What is the justification for restricting genetic analysis to only individuals with European ancestry? What does this mean for applying the calculations of PRS to non-European individuals? This should at the very least be discussed as a limitation to generalizability but warrants a thorough justification in the methods.

14) In the statistical analysis, you mention that Datasets 1-4 were used, but there were only Datasets 1-3 (as I stated before, this is confusing because there are technically 6 datasets…). Please correct this.

15) You mention the covariates to be used but give no reason why these variables were selected. Please give the reasons for each.

RESULTS:
16) There was a fairly high number of participants who recovered from chronic pain in the discovery dataset – over 50%. What do you make of this? Is this in line with other studies? How could this have influenced the results?

17) I would report the supplementary flowcharts in the methods rather than the results.

18) I don't think I would call a p-value of .083 "marginal" (last sentence in the last paragraph of the "Associations between neuroticism/CMP PRSs and CMP Onset/Recovery" section)

19) The way you wrote the results for the mediation analysis makes it seem as though this was a post hoc analysis. If this is the case, please make this clear.

20) You say that there was a significant association between neuroticism score and pain intensities but do not say the direction. Please clarify this.

21) Did you test for any significant differences in your variables of interest between cohorts to validate they were similar enough?

DISCUSSION:

22) The first paragraph fails to report the interesting null findings (similar to the abstract). I would consider including this.

23) In fact, throughout the discussion, there is little to no interpretation of these null findings – I think it is very intriguing why the CMP PRS were not related to the intensity scores yet the neuroticism PRS were. What could this mean? Why wouldn't we see a relationship between intensity and genetic pain risk?

24) What does it mean for recovery that neuroticism was NOT a mediator between CMP PRS and CMP recovery, but was for onset? What would this mean clinically?

25) The beta values reported for the mediation analysis are incredibly small. There is no discussion of the implications of this. It makes me wonder whether this is truly clinically relevant?

26) You state that you "observed an early increase in the bilateral amygdala grey matter volume among healthy participants who later developed CMP…" but is this entirely true? There was an average of I believe 10 years between TP1 and TP3 for the discovery cohort. As you state in the limitations, you cannot account for when during that time period pain first began. It could be co-occurring. I would rephrase this sentence.

TABLES:

27) Table 1- It might be more interpretable to report physical activity as hours per week. As a side note, that seems like a lot of hours each week…

28) Table 1 - the formatting for the education makes it difficult to read.

29) Table 1 – it might be worthwhile to indicate below the table (or in the text) what the TDI score means.

30) Table 1 – the neuroticism scores seem fairly low if reported as a score out of 12. Is that expected? Could this have influenced the results?

31) Table 2 – there is an extra space before each "Dataset 2." Please remove this.

32) Table 3 – it might be easier to following if there were a line delineating between each model for Dataset 1 and 2 within each group (onset/recovery). This could also be fixed by just removing the "outcome variable" column, which I don't think is entirely necessary.

33) No comments for Table 4.

34) Table 5 – similar to my comment for table 3, it would be easier to follow if there were a line to delineate each grouping (a line under "Number of CMP sites" for Neuroticism Score and Neuroticism PRS for both onset and recovery groups).

FIGURES:

35) Figure 1- I think the use of "baseline to follow-up" is misleading given that each dataset uses different time points. It also makes it seem as though there were only 2 visits. I would suggest changing this.

36) Figure 2 – I am not sure what the 1.1% next to "mediation effect" is reflecting. Otherwise, I appreciate this very clear depiction of mediation!

SUPPLEMENTARY:

37) As stated before, you should move the inclusion/exclusion criteria to the main body of the text.

38) It seems that the pain intensity and pain sites phenotypes is exactly the same as in the body of the text? Is this an erorr?

39) For Tables S3 and S4, see my comments for Table 1.

Version 1:

Reviewer comments:

Reviewer #1

(Remarks to the Author)
This revision addressed the concerns raised in the previous round.

Minor suggestions/comments:
(1) I am not sure what is added by reiterating the entire 12 items of neuroticism scale while it has been used as an established scale. I suggest moving them to Supplementary Information.
(2) While the authors claimed similarly modest effect size with two previous studies. They are not comparable to the present findings. Atanassova et al reported just effect sizes, not p-values; Kadimpati et al's p-values are substantially higher than the p-value of this study.

Reviewer #3

(Remarks to the Author)
This study is of good quality. The manuscript is clearly written, and the data are well presented. I have only several concerns for the authors to address:
1. In the baseline TP1–TP4 cohorts, the definition of "pain-free"seems relatively strict, as individuals with either acute pain within the past month or chronic pain were excluded. However, in the PQ stage (after 2019), due to questionnaire limitations, the classification of pain-free participants was based on the following question: "Are you troubled by pain or discomfort, either constantly or intermittently, that has persisted for more than 3 months?" (field 120019). Participants who answered "No" were classified as pain-free. While this question can effectively exclude individuals with chronic pain, it does not rule out participants who might be experiencing acute or subacute pain lasting less than 3 months. Therefore, using this single question to define a "pain-free" population may not be entirely appropriate. Since this criterion appears to have been applied across multiple validation cohorts in the manuscript, it may introduce a potential discrepancy in the clinical definition of the control group. In particular, individuals with acute or subacute pain could be misclassified as pain-free controls, which may lead to misclassification bias and potentially affect the study results. The authors may consider clarifying this definition and discussing its potential impact. If possible, additional filtering criteria or sensitivity analyses would help enhance the robustness of the conclusions.
2. The authors opted to exclude participants with conditions related to anxiety and depression. This decision may facilitate the isolation of neuroticism's effects and mitigate potential confounding due to somatization symptoms. However, it also introduces a notable limitation. In clinical settings, individuals exhibiting high levels of neuroticism frequently present with comorbid anxiety or depression. Consequently, excluding these individuals could result in an artificial truncation of the upper spectrum of neuroticism scores, thereby diminishing the cohort's representativeness. Furthermore, anxiety and depression are prevalent comorbidities within populations experiencing chronic pain. Thus, this selection strategy may constrain the clinical generalizability of the findings, warranting further discussion.
3. The study encompassed participants experiencing pain in the back, hip, knee, or neck, while excluding individuals with widespread pain, non-musculoskeletal pain, psychiatric or neurological disorders, and diabetes. Nevertheless, it remains uncertain whether prevalent inflammatory or autoimmune musculoskeletal conditions, such as Rheumatoid Arthritis or Ankylosing Spondylitis, were accounted for in the exclusion criteria. These conditions are relatively common within clinical populations and may manifest as chronic musculoskeletal pain. If not properly excluded, such cases could inadvertently be included in the chronic musculoskeletal pain (CMP) cohort, potentially introducing heterogeneity and compromising the reliability of the study's findings. The authors are encouraged to elucidate how these conditions were addressed in the cohort definition.
4.In reference to Figure S2 (Study Sample Flowchart), there exists a significant logical inconsistency within the exclusion criteria that undermines the validity of the study sample selection process. Specifically, the authors delineate an exclusion of participants experiencing acute or chronic pain (criterion iv), yet the final sample at Time Point 1 is characterized as comprising individuals with chronic musculoskeletal pain (N=51,577).
5. The authors claim no dataset overlap, but they don't clearly explain how patients eligible for multiple datasets are assigned. The supplementary material shows the validation cohort excludes patients from the discovery cohort, giving the latter priority. This could lead to selection bias, leaving the validation cohort with a non-representative sample and weakening its statistical power and external validity. The authors need to clarify the assignment rule for overlapping patients and discuss how this cohort design might affect the statistical results, including risks of overfitting and reduced generalizability.
6. The findings indicate that a higher polygenic risk score (PRS) for neuroticism is significantly correlated with an elevated risk of persistent chronic musculoskeletal pain (CMP) in the discovery cohort (Dataset A: TP1–TP3; p=0.023, FDR<0.05, OR=1.082, 95% CI: 1.010–1.160). Conversely, this association did not achieve statistical significance in Dataset B (TP1–PQ; p=0.083, FDR=0.083, OR=1.042, 95% CI: 0.994–1.093), which functions as the primary validation cohort. In light of the absence of statistical significance in the validation dataset, it is advisable for the authors to temper their interpretation of the neuroticism PRS results in the Discussion and Conclusion sections to more accurately reflect the cumulative evidence.
7. The authors identify differences in amygdala gray matter volume between groups in Dataset 3, while no such differences

are evident in Dataset C. Notably, several prior studies have indicated that persistent pain may induce structural changes in the amygdala, such as increased gray matter volume (e.g., PMID: 23823184; 38606278). Consequently, the observed enlargement of the amygdala in the TP3 onset cohort could potentially represent neuroplastic changes secondary to early or unreported pain, rather than serving as a predisposing factor. This interpretation might also account for the absence of differences in Dataset C, where both groups experienced chronic pain at baseline. The authors are encouraged to consider discussing this alternative explanation when interpreting the imaging findings.

Reviewer #4

(Remarks to the Author)
This study examines the relationships between neuroticism, polygenetic risk scores and amygdala grey matter and chronic pain onset and recovery over time. The authors identify positive associations between these variables, concluding that neuroticism and genetic risk factors influence the onset of and recovery from chronic pain in important ways, potentially through the amygdala grey matter volume as a neurobiological mechanism, and suggest these factors may even serve as biomarkers for CPM prevention and management. The use of a large dataset and within-study replications on independent subsets are major strengths of the study, and I appreciate the efforts put into testing the robustness of the findings. However, the small effect sizes and lack of critical discussion of the concrete meaning of these findings leave me unconvinced of the clinical relevance of these results.

Major concerns:
While the findings seem robust, the effect sizes for the mediation effects seem extremely small (0.000086-0.00086), diminishing the clinical relevance of these effects. The authors describe this as moderate/modest while in my opinion it would even be a bit of an overstatement to call them small (in my experience, a mediation effect of ~0.01 is generally already considered small). A critical discussion of the meaning of these effects in concrete terms would be needed to evaluate the clinical value of the results.

On a similar note, the authors do not discuss effect sizes for their other analyses, therefore their clinical relevance is not clear. For example, most of the odds ratios for the logistical regressions also seem rather small (1.025-1.093), but it is not explained how we can interpret these findings. For example, I think with an OR of 1.05, the risk for developing chronic pain is 5% higher when neuroticism is x points higher (compared to lower), but it matters a lot if the baseline risk is 0.1% (add 5% and it is 0.005% higher for people with higher neuroticism) or 20% (add 5% and it is 1% higher). This is especially important when these metrics are presented as potential risk factors and/or biomarkers to be used in clinical practice.

The authors lean into the idea of longitudinal analyses and pain trajectories to aid with causal interpretations. While the time component does add to the relevance of the study, they only use two time points in each analysis and there is little critical discussion of the other major challenge in determining causality, namely the potential for confounding effects. With many years in between measurements, it seems plausible many other factors may have contributed (much more?) to the development and/or recovery of chronic pain. And there may have been other factors that contribute to both neuroticism and chronic pain, but were not included in the analysis.

Other concerns:
In the results (line 323-324) it is reported that the relationship between neuroticism and CPM persistence is replicated in both validation datasets, however, in Table 2 the results for dataset C seem to be non-significant?

While large and rich datasets like the UK Biobank offer many strengths and advantages, one potential pitfall is the risk of decisions on which data to use and how being influenced by prior knowledge of the data, which makes careful consideration and decision making prior to data analysis (ideally before seeing the data) even more important. Which makes me wonder, were the analyses preregistered prior to analysis? Were any analyses added at a later stage? This would be good to mention in the manuscript for transparency.

The authors mention that analyses were restricted to people of European ancestry, and acknowledge this in the limitations of the study. This is essential context for the study results, which would be important to mention in the title or else in the abstract.

The exclusion criteria are quite strict. On one hand, this can help reduce the impact of potential confounding factors. On the other hand, this makes generalization to the general population difficult, especially as chronic pain is often comorbid with other mental and physical health conditions. Many people also experience disability due to chronic pain, does the exclusion criteria relating to disability (line 138) mean they were also excluded from the study? If so, you would get an even more skewed representation of the population of people experiencing chronic pain. These issues would be good to reflect on in the discussion.

Version 2:

Reviewer comments:

Reviewer #3

(Remarks to the Author)

All my concerns have been addressed. Hence, I would like to recommend this revised manuscript for acceptance.

Reviewer #4

(Remarks to the Author)
I thank the authors for their responses, and find the concerns I raised sufficiently addressed.

Open Access This Peer Review File is licensed under a Creative Commons Attribution 4.0 International License, which permits use, sharing, adaptation, distribution and reproduction in any medium or format, as long as you give appropriate credit to the original author(s) and the source, provide a link to the Creative Commons license, and indicate if changes were made.
In cases where reviewers are anonymous, credit should be given to 'Anonymous Referee' and the source.
The images or other third party material in this Peer Review File are included in the article’s Creative Commons license, unless indicated otherwise in a credit line to the material. If material is not included in the article’s Creative Commons license and your intended use is not permitted by statutory regulation or exceeds the permitted use, you will need to obtain permission directly from the copyright holder.
To view a copy of this license, visit https://creativecommons.org/licenses/by/4.0/

Response to reviewer's comments:

We sincerely thank the reviewers for their constructive and insightful comments. Please find below our detailed point-by-point responses. We would be pleased to make further revisions if requested.

Reviewer #1:

This is a very well-written manuscript. Overall structure and organization of study findings are excellent. UK Biobank datasets are a well-established and tested dataset. The examination of behavioral and genetic factors in pain longitudinally seems to be a major contribution of the study.

We thank the reviewer for the thoughtful comments and suggestions.

1. However, I have a reservation about the novelty of the study findings. Previous studies have noted the relationship between neuroticism and pain, although it is difficult to find the directionality of the relationship. This study reports those relationships over different time points. The main 'novel' finding seems to be the mediating role of neuroticism on the relationship between CMP PRS and CMP onset. To me, the results may not be sufficient to make such conclusion statistically, in addition to the null findings from the recovery cohort. The result of uncorrected $p < .03$ does not seem to reflect substantial evidence for the role of the mediator variable.

Response: We agree with the reviewer that the association between neuroticism and pain has been well documented in previous studies. Our study extends this literature by addressing a key limitation—testing the *directionality* of this relationship using a prospective design. Leveraging the multipoint longitudinal dataset from the UK Biobank, our findings indicate that higher levels of neuroticism are prospectively associated with both the onset and the recovery of chronic pain. To our knowledge, few prior studies have examined this relationship using a similar prospective framework.

Also, if we understand correctly, the reviewer suggests that when including both onset and recovery analyses, the corrected significance threshold should be adjusted to $p = 0.025$ (i.e., 0.05/2), and therefore a $p = 0.03$ would not be considered significant. We would first like to clarify that, consistent with all our analyses, we have revised the manuscript to use the discovery dataset to test the primary hypothesis, with the validation dataset serving to confirm the findings derived from the discovery analysis. Accordingly, we conducted the mediation analysis using the discovery dataset, which yielded a significant result ($p = 0.014$; see Figure 2 for details) after p-value adjustment. The validation dataset was used solely for replication purposes and therefore does not require p-value correction. We apologize for not initially performing this mediation analysis using the discovery cohort, as our original goal was to maximize the sample size by using only Dataset 2 and have revised the manuscript accordingly.

2. In Figure 2, the direct effect of the relationship between CMP PRS and onset almost explains the total effect of the relationship between them, while the effects are relatively

small, assuming betas are standardized effect sizes. The size of the mediation effect is even tinier. Considering the sample sizes of the study, it is not unreasonable not to exclude the possibility of a false positive. Separate associations among variables are also reported. However, those associations do not necessarily make convincing evidence for the mediating effects of neuroticism on pain, as claimed in the discussion and the abstract. More substantial data to back up the mediation effect of neuroticism could be helpful to convince readers about the novel findings of this study.

Response: We thank the reviewer for the thoughtful comments. As outlined above, we have revised the manuscript to include an additional mediation analysis using the discovery cohort (Dataset 1) and subsequently used the validation dataset (Dataset 2) to confirm our findings. This replication strengthens the evidence for the mediation effect and reduces the likelihood that the original result was a false positive. Both analyses yielded similar results, which we hope will help convince readers of the robustness and novelty of our findings.

We also agree with the reviewer that the effect sizes observed in both analyses are small. However, our results are consistent with previous literature on neuroticism and pain. For example, a recent meta-analysis (Atanassova, Dimana V., et al., 2024) reported a significant but modest correlation between neuroticism and pain tolerance ($r = -0.07$), with several studies reporting similarly small yet significant effects. Likewise, in a prior study (Kadimpati, Sandeep, et al., 2015), neuroticism was significantly associated with pain catastrophizing, explaining approximately 1% of the variance ($Sr^2 = 0.01$; $p = 0.004$).

These findings underscore that neuroticism, while a modest contributor, is a consistent psychological factor influencing pain perception. Thus, although the observed effects are small, they provide an important foundation for future research aimed at elucidating the role of personality traits in shaping pain outcomes at the population level.

We have updated the *Results* section and Tables to include the additional mediation analyses and have revised the manuscript to reduce the emphasis on these findings, given the small observed effect size. The limitations have been explicitly discussed in the revised *Discussion* section, along with supporting references. Finally, we have clarified in the caption of Figure 2 that the reported beta coefficients were derived from standardized (z-score normalized) variables.

References:

ATANASSOVA, Dimana V., et al. Unpacking the relationship between Big Five personality traits and experimental pain: a systematic review and meta-analysis. *Neuroscience & Biobehavioral Reviews*, 2024, 163: 105786.

KADIMPATI, Sandeep, et al. Associations between neuroticism and depression in relation to catastrophizing and pain-related anxiety in chronic pain patients. *PloS one*, 2015, 10.4: e0126351.).

Reviewer #2:

This study explores the role of neuroticism, genetic risk, and amygdala structure in the development and recovery of chronic musculoskeletal pain (CMP) using longitudinal data from the UK Biobank of those who did and did not have CMP at baseline as well as those who did and did not recover at follow-up. The authors report significant evidence for genetic and causal relationships between neuroticism and CMP onset and recovery, supporting a neurobiological mechanism linking psychological states to chronic pain vulnerability. This is an impressive manuscript with important implications for the field.

Response: We thank the reviewer for the thoughtful comments and suggestions.

1. Overall, I think the abstract succinctly sums up the results and importance of the paper. I would encourage the authors to include some of the null findings as well (neuroticism did not mediate CMP PRS-CMP recovery; CMP PRS was not associated with pain intensity phenotypes, amygdala volume related to neuroticism in CMP onset participants but not CMP recovery), as this could be of interest to individuals skimming the abstract.

Response: We thank the reviewer for this suggestion. We have inserted the information on the null findings in the 'Results' section of the abstract.

2. I think there needs to be a more thorough discussion of the background of the overlapping biological mechanisms. As it stands, they are convincing but too vague – for example, are there specific genes that are related to both neuroticism and CMP? Have people theorized as to what these genetic mechanisms mean for both neuroticism and CMP? What about the purported structural and functional changes you mention – you say that these are associated with both neuroticism and CMP but don't discuss the direction (greater connectivity, less volume, etc)? I would like to see more concrete evidence to support what you are saying.

Response: We have included more information in the Introduction section on the overlapping biological mechanisms linking chronic pain and neuroticism. Regarding the structural and functional changes, we added details to further explain the association between the amygdala and both neuroticism and CMP.

3. You seem to suggest that there is already a plethora of work linking neuroticism and pain perception and persistence, but only cite one paper. I'd like to see more work cited/discussed to support this tone.

Response: We have added additional references in the *Introduction* to further support the link between neuroticism and pain perception as well as further discussion on this topic.

4. This may have more to do with the methods, but what is the justification for only using CMP participants? Why disregard other types of chronic pain? Do you think there is something unique

about the relationship between neuroticism and CMP vs. neuroticism and chronic pain in general? If so, a much more detailed explanation is required in the introduction.

Response: We thank the reviewer for the thoughtful question. We restricted our analysis to chronic musculoskeletal pain (CMP) to reduce heterogeneity and improve interpretability of the results. Chronic pain is a broad construct encompassing diverse conditions, each with distinct etiologies and pathophysiological mechanisms. For example, facial pain, headache, and abdominal pain often reflect neurological, vascular, or gastrointestinal processes, which differ substantially from musculoskeletal mechanisms.

Including such heterogeneous pain types could obscure the specific pathways linking psychological traits, such as neuroticism, to pain susceptibility. Focusing on CMP is particularly justified because recent genetic studies (TSEPILOV, Yakov A., et al.) using genetically independent phenotypes have demonstrated that CMP shares a common genetic component. Importantly, the primary shared genetic component showed strong enrichment for central nervous system pathways and exhibited significant genetic correlations with neuroticism and other psychiatric traits, providing direct evidence that CMP and neuroticism are linked through overlapping biological mechanisms. By concentrating on CMP, one can therefore more precisely investigate these convergent neurobiological pathways and their contribution to the interplay between personality traits and pain risk, while minimizing confounding from unrelated pain etiologies.

We have introduced this concept in the introduction by highlighting recent genetic evidence specifically linking CMP and neuroticism.

References:

TSEPILOV, Yakov A., et al. Analysis of genetically independent phenotypes identifies shared genetic factors associated with chronic musculoskeletal pain conditions. Communications biology, 2020.

5. Overall, I felt the methods were difficult to follow given the complexity of the sample used. It was not until I examined the flowcharts in the supplementary that I full understood how each dataset was created (regarding which participants were selected for which dataset and why). I think it would be incredibly beneficial if you included a timeline figure to include each visit, the dates, which variable that you are using were collected at each visit, etc. This could be included in Figure 1 as Part A or B or stand alone as its own figure. Delineating which study visits were used for which cohorts and the final N for each would be beneficial here.

Response: We agree with the reviewer and have modified Figure 1 to improve clarity. We inserted a timeline for data collection, variable definitions, and the datasets building process, including the final N used for the analysis. Supplementary Flowcharts of datasets building complement the information contained in the new Figure 1.

6. You should report the exclusion criteria in the main body of the paper and not the supplementary materials.

Response: We have moved the inclusion/exclusion criteria section to the main body of the manuscript.

7. What is the justification for using the study visits selected? I know that PQ was selected because it was the only visit that reported pain intensities (this is noted under the pain intensity and pain sites phenotypes section of the methods but should be made clearer much earlier). But why not use TP2 at all? I think there is a need for a stronger discussion as to why you used each visit for each of the datasets.

Response: The justification for selecting the study visits was based on participant numbers. TP1 (baseline assessment), TP3, and PQ (a special online assessment) included the largest number of participants. Although TP2 also contained relevant information, attendance was low. The subset of individuals with both TP1 (baseline) and TP2 data was especially small, resulting in a dataset that was underpowered for robust analyses. This limitation was further compounded by the need to avoid sample overlap between cohorts, which left very few participants with TP2 data available outside the first discovery cohort. We have inserted this information in the materials and methods (section 'CMP onset and recovery datasets').

8. Relatedly, what if a participant reported pain starting at TP2, pain free again by TP3 but developed pain again by PQ? How would you classify that participant? Such instances could be made clearer.

Response: Regarding the potential change of pain condition from TP 3 to PQ, our exclusion criteria—specifically, no overlapping participants across datasets—account for this possibility. Dataset 1 (TP 1 to TP 3) and Dataset 2 (TP 1 to PQ) included two independent cohorts with no participant overlap. Therefore, any participant in Dataset 2 who also had a TP 3 assessment would have been excluded, ensuring that no individual could appear in both datasets and preventing changes in pain status from TP 3 to PQ within the analyzed samples.

As for TP 2, it represents a very small subset of participants. The initial sample of ~20,000 at TP 2 was further reduced by excluding individuals with ICD-9 or ICD-10 diagnoses, assigning phenotypes (CMP or pain-free), and screening out participants with acute pain or coexisting pain types. Consequently, the number of CMP and pain-free individuals at TP 2 is limited. Nevertheless, following the reviewer's suggestion, we checked Datasets 1 and 2 (as well as subsets A and B) for participants whose condition changed at the intermediate TP 2 assessment.

In Dataset 1, we found 64 participants (0.6% of the sample) who were pain-free at TP 1, CMP at TP 2, and pain-free again at TP 3. These individuals were classified as steady pain-free in Dataset 1. Similarly, in Dataset A, we found 35 participants (1.01% of the sample) who were CMP at TP 1, pain-free at TP 2, and CMP again at TP 3. These participants were classified as persistent CMP in Dataset A.

Given their small number, we believe this assumption does not meaningfully affect the results.

9. Although I appreciate wanting to maintain the consistency for Datasets 1-3, I found it a bit confusing given that it is technically 6 different datasets that are separated by whether they are looking at onset or recovery as the main outcome of interest. Perhaps it might be worthwhile to rename it as Datasets 1-3 and corresponding Datasets A-C? It maintains the similarity that you initially wanted and makes it clearer that these are totally different datasets. Just a suggestion.

Response: We appreciate the suggestion and believe that the proposed renaming will improve clarity. We renamed the onset and recovery datasets differently and updated the manuscript, as well as relevant Tables/Figures.

10. Did you consider that some individuals that reported having pain "all over" could have fibromyalgia, a condition of widespread musculoskeletal pain? What is the advantage of excluding these participants?

Response: Because the etiology of general pain in the UK Biobank is unknown and could reflect a variety of underlying conditions, we could not reliably classify these participants as fibromyalgia or any other specific disorder. To avoid introducing heterogeneity or bias into our analyses, we therefore excluded participants reporting general pain.

11. Is the neuroticism survey a validated survey?

Response: Yes, the neuroticism survey we used is a validated instrument. Specifically, it is a 12-item version of the Eysenck Personality Inventory Neuroticism scale (EPIN-R). We have added a reference in the manuscript.

12. I wonder what is the purpose of calculating a sum of pain intensities across each pain site. Is this truly the best approach to capture somebody having "more" pain?

Response: The purpose of calculating the sum of pain intensities across different pain sites in our exploratory analysis was to capture both the extent and the severity of pain. By summing the intensities, we aimed to identify individuals experiencing more widespread pain across multiple sites as well as higher overall pain levels, allowing us to explore potential associations between neuroticism and a broader pain burden. While this approach is exploratory and it might not be the best approach to capture participants with more pain, it provides a simple way to quantify cumulative pain that reflects both number of affected sites and intensity at each site.

13. What is the justification for restricting genetic analysis to only individuals with European ancestry? What does this mean for applying the calculations of PRS to non-European individuals? This should at the very least be discussed as a limitation to generalizability but warrants a thorough justification in the methods.

Response: We restricted analyses to individuals of European ancestry to minimize population stratification bias, as LD structure and allele frequencies differ across ancestries. About 94% of

UK Biobank participants are of European ancestry, which also explains our focus. However, this limits the generalizability of PRS to non-European populations, where predictive accuracy might be reduced. We have added this discussion to the limitations section.

14. In the statistical analysis, you mention that Datasets 1-4 were used, but there were only Datasets 1-3 (as I stated before, this is confusing because there are technically 6 datasets…). Please correct this.

Response: We apologize for the mistake and have corrected the sentence in the statistical analysis section, ensuring to mention both onset and recovery datasets.

15. You mention the covariates to be used but give no reason why these variables were selected. Please give the reasons for each.

Response: We have inserted references to support the selection of the covariates and briefly explained our choice in the methods.

16. There was a fairly high number of participants who recovered from chronic pain in the discovery dataset – over 50%. What do you make of this? Is this in line with other studies? How could this have influenced the results?

Response: The relatively high number of participants who recovered from chronic pain in the discovery dataset—over 50%—may partly reflect the follow-up period, which allowed sufficient time for symptom resolution. While the lack of similar longitudinal characterization prevents direct comparisons with other studies, we do not believe this substantially influenced our results. The availability of intermediate assessments, indeed, could have helped capture changes in pain status and supported the robustness of our findings despite the observed recovery rate. Furthermore, we believe that the fact that both the CMP and pain-free groups included substantial numbers of participants likely did not bias the results towards one class, which is preferable to having a strongly imbalanced class distribution.

17. I would report the supplementary flowcharts in the methods rather than the results.

Response: We have moved the information on the supplementary flowcharts to the methods section to improve clarity.

18. I don’t think I would call a p-value of .083 “marginal” (last sentence in the last paragraph of the “Associations between neuroticism/CMP PRSs and CMP Onset/Recovery” section)

Response: We have changed the sentence to ‘no significant associations’.

19. The way you wrote the results for the mediation analysis makes it seem as though this was a post hoc analysis. If this is the case, please make this clear.

Response: Mediation analysis did not represent a post-hoc analysis. We have rephrased the results section to avoid misunderstanding.

20. You say that there was a significant association between neuroticism score and pain intensities but do not say the direction. Please clarify this.

Response: We have clarified the direction of this association.

21. Did you test for any significant differences in your variables of interest between cohorts to validate they were similar enough?

Response: We tested for differences in our variables of interests between datasets and inserted the results in the manuscript, in addition to describing the procedure in the statistical analysis section.

DISCUSSION:

22. The first paragraph fails to report the interesting null findings (similar to the abstract). I would consider including this.

Response: We have added the null findings in the first paragraph of the Discussion section.

23. In fact, throughout the discussion, there is little to no interpretation of these null findings – I think it is very intriguing why the CMP PRS were not related to the intensity scores yet the neuroticism PRS were. What could this mean? Why wouldn't we see a relationship between intensity and genetic pain risk?

Response: We have discussed the null findings in relation to the association between CMP PRS and intensity scores by stating that 'higher PRS for CMP is significantly associated with CMP outcomes, including both onset and recovery, though it was not significantly associated with pain intensity phenotypes, indicating that the genetic factors underlying chronic pain susceptibility may differ from those influencing pain severity'. We may speculate that the genetic mechanisms underlying chronic musculoskeletal pain susceptibility may, at least in part, differ from those of pain perception.

24. What does it mean for recovery that neuroticism was NOT a mediator between CMP PRS and CMP recovery, but was for onset? What would this mean clinically?

Response: This finding suggests that neuroticism primarily acts as a vulnerability factor that amplifies genetic risk for developing chronic pain in otherwise healthy individuals. Once CMP

has already manifested, however, the pathways linking genetic risk to recovery are likely influenced by other biological, psychological, or environmental factors, and the mediating role of neuroticism in recovery appears limited.

25. The beta values reported for the mediation analysis are incredibly small. There is no discussion of the implications of this. It makes me wonder whether this is truly clinically relevant?

Response: We thank the reviewer for the comments. We agree that only a moderate effect size observed. However, the findings of modest effects of the neuroticism on pain are in line with previous literature reviewer (ATANASSOVA, Dimana V., et al.) (KADIMPATI, Sandeep, et al.), where neuroticism was found to be a consistent but modest influencing factor for pain. Further research might elucidate this relationship.

References:

ATANASSOVA, Dimana V., et al. Unpacking the relationship between Big Five personality traits and experimental pain: a systematic review and meta-analysis. *Neuroscience & Biobehavioral Reviews*, 2024, 163: 105786.

KADIMPATI, Sandeep, et al. Associations between neuroticism and depression in relation to catastrophizing and pain-related anxiety in chronic pain patients. *PloS one*, 2015, 10.4: e0126351.).

26. You state that you “observed an early increase in the bilateral amygdala grey matter volume among healthy participants who later developed CMP…” but is this entirely true? There was an average of I believe 10 years between TP1 and TP3 for the discovery cohort. As you state in the limitations, you cannot account for when during that time period pain first began. It could be co-occurring. I would rephrase this sentence.

Response: There may have been a misunderstanding regarding our approach for detecting early increases in amygdala grey matter volume. For this analysis, both pain-free phenotypes and amygdala volumes were extracted at TP3, which represents the first assessment with imaging data available. Follow-up conditions were assessed using TP4, with an average follow-up period of three years. Pain-free participants at TP3 were categorized based on their TP4 status (steady pain-free vs. CMP onset). These two groups showed significant differences in amygdala grey matter volume at TP3. We have rephrased this description to avoid potential misunderstanding.

TABLES:

27. Table 1- It might be more interpretable to report physical activity as hours per week. As a

side note, that seems like a lot of hours each week…

Response: We have changed the physical activity as hours per week in Table 1 and in all the relevant tables as suggested. The reported physical activity, as also specified in Table S2, represents the Total Metabolic Equivalent Task (MET) minutes per week for all activity including walking and moderate and vigorous activity. It may seem like a lot of hours because it incorporates different aspects of movement/activity.

28. Table 1 - the formatting for the education makes it difficult to read.

Response: We have re-formatted the education section in Table 1 and in all the other tables reporting education scores to improve readability.

29. Table 1 – it might be worthwhile to indicate below the table (or in the text) what the TDI score means.

Response: We have added details on the TDI score below each table as suggested.

30. Table 1 – the neuroticism scores seem fairly low if reported as a score out of 12. Is that expected? Could this have influenced the results?

Response: Yes, the relatively low neuroticism scores are expected, because we excluded participants with a wide range of psychiatric, neurological, and related medical conditions. In light of the consideration that the mean and SD reported across all UK Biobank participants—including those with documented psychological disorders—is around 4 (see UK Biobank Field 20127), the lower scores in our healthy controls are reasonable.

31. Table 2 – there is an extra space before each "Dataset 2." Please remove this.

Response: We have removed the space after Dataset 2 in Table 2 as suggested.

32. Table 3 – it might be easier to following if there were a line delineating between each model for Dataset 1 and 2 within each group (onset/recovery). This could also be fixed by just removing the "outcome variable" column, which I don't think is entirely necessary.

Response: We have reformatted Table 3 by removing the 'outcome variable' column to make it easier to follow each model/dataset.

33. No comments for Table 4.

----

34. Table 5 – similar to my comment for table 3, it would be easier to follow if there were a line to delineate each grouping (a line under “Number of CMP sites” for Neuroticism Score and Neuroticism PRS for both onset and recovery groups).

Response: We have reformatted Table 5 by adding a line to delineate each different grouping as suggested.

FIGURES:

35. Figure 1- I think the use of “baseline to follow-up” is misleading given that each dataset uses different time points. It also makes it seem as though there were only 2 visits. I would suggest changing this.

Response: We have reformatted Figure 1, which now includes specific information on each timepoint used for each dataset.

36. Figure 2 – I am not sure what the 1.1% next to “mediation effect” is reflecting. Otherwise, I appreciate this very clear depiction of mediation!

Response: We have modified Figure 2, due to the additional mediation analyses performed.

SUPPLEMENTARY:

37. As stated before, you should move the inclusion/exclusion criteria to the main body of the text.

Response: We have moved the inclusion/exclusion criteria section in the main body of the manuscript as recommended.

38. It seems that the pain intensity and pain sites phenotypes is exactly the same as in the body of the text? Is this an erorr?

Response: We apologize for the mistake and have removed the paragraph from the Methods, leaving the description of these data in the Supplementary Materials.

39. For Tables S3 and S4, see my comments for Table 1.

Response: As in our response for Table 1, the relatively low neuroticism scores are expected, because we excluded participants with a wide range of psychiatric, neurological, and related medical conditions. In light of the consideration that the mean and SD reported across all UK

Biobank participants—including those with documented psychological disorders—is around 4 (see UK Biobank Field 20127), the lower scores in our healthy controls are reasonable.

Point-by-point responses

We thank the editor for the opportunity to revise the manuscript and the reviewers for their constructive and insightful comments. Please find below our detailed point-by-point responses. We would be pleased to make further revisions if requested.

Reviewer #1 (Remarks to the Author):

This revision addressed the concerns raised in the previous round.

Minor suggestions/comments:
(1) I am not sure what is added by reiterating the entire 12 items of neuroticism scale while it has been used as an established scale. I suggest moving them to Supplementary Information.

Response: We agree and have moved the full list of the 12 neuroticism items to the Supplementary Information section (Table S2), while keeping a concise description in the main text.

(2) While the authors claimed similarly modest effect size with two previous studies. They are not comparable to the present findings. Atanassova et al reported just effect sizes, not p-values; Kadimpati et al's p-values are substantially higher than the p-value of this study.

Response: We thank the reviewer for this significant clarification. We have revised the Discussion to more carefully contextualize effect sizes by adding the following: "Direct comparisons across studies remain limited by differences in outcome definitions and reporting metrics."

Reviewer #3 (Remarks to the Author):
Overall, I find the authors' revisions and rebuttals to be satisfactory, and they have addressed reviewer #2's comments appropriately. This study is of good quality. The manuscript is clearly written, and the data are well presented. I have only several additional concerns for the authors to address:

1. In the baseline TP1–TP4 cohorts, the definition of "pain-free"seems relatively strict, as individuals with either acute pain within the past month or chronic pain were excluded. However, in the PQ stage (after 2019), due to questionnaire limitations, the classification of pain-free participants was based on the following question: "Are you troubled by pain or discomfort, either constantly or intermittently, that has persisted for more than 3 months?" (field 120019). Participants who answered "No" were classified as pain-free. While this question can effectively exclude individuals with chronic pain, it does not rule out participants who might be experiencing acute or subacute pain lasting less than 3 months. Therefore, using this single question to define a "pain-free" population may not be entirely appropriate. Since this criterion appears to have been applied across multiple validation cohorts in the manuscript, it may introduce a potential discrepancy in the clinical definition

of the control group. In particular, individuals with acute or subacute pain could be misclassified as pain-free controls, which may lead to misclassification bias and potentially affect the study results. The authors may consider clarifying this definition and discussing its potential impact. If possible, additional filtering criteria or sensitivity analyses would help enhance the robustness of the conclusions.

Response: We have clarified the definition of "pain-free" across timepoints in the Methods section. The pain-free control and CMP cohorts were defined based on the following question: "In the last month have you experienced any of the following that interfered with your usual activities (You can select more than one answer)?" (extracted using UK Biobank data field 6159). Possible choices to the questions were: (1) "headaches," (2) "facial pain," (3) "neck or shoulder pain," (4) "back pain," (5) "stomach or abdominal pain," (6) "hip pain," (7) "knee pain," (8) "pain all over the body," (9) "none of the above," and (10) "prefer not to answer". If (8), (9) or (10) were selected, no additional choices were required. Participants who answered (9) were assigned to the pain-free group, while those who selected (8) or (10) were excluded from the analysis. We have clarified this point in the manuscript.

2. The authors opted to exclude participants with conditions related to anxiety and depression. This decision may facilitate the isolation of neuroticism's effects and mitigate potential confounding due to somatization symptoms. However, it also introduces a notable limitation. In clinical settings, individuals exhibiting high levels of neuroticism frequently present with comorbid anxiety or depression. Consequently, excluding these individuals could result in an artificial truncation of the upper spectrum of neuroticism scores, thereby diminishing the cohort's representativeness. Furthermore, anxiety and depression are prevalent comorbidities within populations experiencing chronic pain. Thus, this selection strategy may constrain the clinical generalizability of the findings, warranting further discussion.

ResponseWe agree with the reviewer and have expanded the Limitations section accordingly. We have added the following: "Our study excluded participants with anxiety, depression, long-term disability, and related conditions to isolate neuroticism-specific effects and reduce confounding factors. However, we acknowledge that these are prevalent comorbidities within populations experiencing chronic pain, and their exclusion may have constrained the clinical generalizability of our findings to the broader chronic pain population."

3. The study encompassed participants experiencing pain in the back, hip, knee, or neck, while excluding individuals with widespread pain, non-musculoskeletal pain, psychiatric or neurological disorders, and diabetes. Nevertheless, it remains uncertain whether prevalent inflammatory or autoimmune musculoskeletal conditions, such as Rheumatoid Arthritis or Ankylosing Spondylitis, were accounted for in the exclusion criteria. These conditions are relatively common within clinical populations and may manifest as chronic musculoskeletal pain. If not properly excluded, such cases could inadvertently be included in the chronic musculoskeletal pain (CMP) cohort, potentially introducing heterogeneity and

compromising the reliability of the study's findings. The authors are encouraged to elucidate how these conditions were addressed in the cohort definition.

Response: We have clarified in the Methods and Limitations sections that the exclusion criteria based on ICD-9/10 codes include a broad range of medical conditions, which encompass relevant inflammatory and autoimmune disorders where applicable.

The following text was added to the Methods: "Notably, the ICD-9/10 codes used for our exclusion criteria encompass relevant inflammatory and autoimmune musculoskeletal conditions (including Rheumatoid Arthritis and Ankylosing Spondylitis), which are common in clinical populations and may manifest as CMP. Despite the scope of these exclusion codes, we acknowledge that some residual heterogeneity may remain and influence our findings."

The following was added to the Limitations section: "Moreover, while our exclusion criteria using the ICD-9/10 codes accounted for a wide range of inflammatory and autoimmune musculoskeletal conditions, we acknowledge that residual heterogeneity may remain and could influence our results."

4.In reference to Figure S2 (Study Sample Flowchart), there exists a significant logical inconsistency within the exclusion criteria that undermines the validity of the study sample selection process. Specifically, the authors delineate an exclusion of participants experiencing acute or chronic pain (criterion iv), yet the final sample at Time Point 1 is characterized as comprising individuals with chronic musculoskeletal pain (N=51,577).

Response: We thank the reviewer for identifying this issue. We have revised Figure S2 and clarified the description to distinguish between exclusion criteria. We note that only "those with (i) psychological or neurological disorders, as identified by ICD-10 or ICD-9 diagnosis, (ii) a diagnosis of diabetes, and (iii) long-standing illness, disability or infirmity" were excluded in the initial filtering process.

5. The authors claim no dataset overlap, but they don't clearly explain how patients eligible for multiple datasets are assigned. The supplementary material shows the validation cohort excludes patients from the discovery cohort, giving the latter priority. This could lead to selection bias, leaving the validation cohort with a non-representative sample and weakening its statistical power and external validity. The authors need to clarify the assignment rule for overlapping patients and discuss how this cohort design might affect the statistical results, including risks of overfitting and reduced generalizability.

Response: We have clarified in the Methods that participants eligible for multiple datasets were assigned to only one cohort, with priority given to discovery cohorts to preserve independence of validation datasets.

We added the following text to the Methods: "The discovery cohorts (Dataset 1 and A) were given priority during the assignment process to preserve sample size and statistical power for the primary analysis. Participants assigned to the discovery cohorts were subsequently excluded from all validation cohorts, ensuring no overlap between the datasets. We

acknowledge that this prioritization strategy may reduce the representativeness and statistical power of validation cohorts and introduce selection bias."

6. The findings indicate that a higher polygenic risk score (PRS) for neuroticism is significantly correlated with an elevated risk of persistent chronic musculoskeletal pain (CMP) in the discovery cohort (Dataset A: TP1–TP3; p=0.023, FDR<0.05, OR=1.082, 95% CI: 1.010–1.160). Conversely, this association did not achieve statistical significance in Dataset B (TP1–PQ; p=0.083, FDR=0.083, OR=1.042, 95% CI: 0.994–1.093), which functions as the primary validation cohort. In light of the absence of statistical significance in the validation dataset, it is advisable for the authors to temper their interpretation of the neuroticism PRS results in the Discussion and Conclusion sections to more accurately reflect the cumulative evidence.

Response: We agree with the reviewer and have revised the Results, Discussion, and Conclusion to temper the interpretation of neuroticism PRS findings. The following text was added to the Results: "However, this association showed no significance in Dataset B, the primary validation cohort (TP 1 – PQ; p = 0.083, FDR = 0.083, OR = 1.042, 95% CI: 0.994 – 1.093). Due to the absence of replication, these findings should be interpreted with caution (Table 3, Table S9)."

The following text was added to the Discussion: "However, while this association demonstrated a significant positive correlation in the discovery cohort (Dataset A; p = 0.023), it did not reach statistical significance in the primary validation cohort (Dataset B; p = 0.083). Due to lack of replication, these findings should be interpreted cautiously."

The following text was added to the Conclusion: "We found associations between genetic risk for neuroticism and CMP onset; however, its role in CMP recovery was less clear."

7. The authors identify differences in amygdala gray matter volume between groups in Dataset 3, while no such differences are evident in Dataset C. Notably, several prior studies have indicated that persistent pain may induce structural changes in the amygdala, such as increased gray matter volume. Consequently, the observed enlargement of the amygdala in the TP3 onset cohort could potentially represent neuroplastic changes secondary to early or unreported pain, rather than serving as a predisposing factor. This interpretation might also account for the absence of differences in Dataset C, where both groups experienced chronic pain at baseline. The authors are encouraged to consider discussing this alternative explanation when interpreting the imaging findings.

Response: We thank the reviewer for this valuable perspective and have incorporated this interpretation into the Discussion as follows: " The presence of amygdala enlargement specifically in individuals who later developed CMP, but not in those already living with chronic pain, suggests that increased amygdala volume may represent a neural risk marker for CMP onset rather than solely a structural consequence of established chronic pain. This interpretation is further supported by the observed association between amygdala volume and neuroticism. These findings are consistent with previous studies reporting increased amygdala volume in chronic pain populations. We extend this literature by suggesting that

amygdala enlargement may precede the development of CMP. However, an alternative interpretation is that individuals in the incident CMP group may have already experienced subthreshold or unreported pain at baseline, which could have contributed to amygdala-related changes. The absence of significant amygdala volume differences in Dataset C, in which all participants had established chronic pain at baseline, may be explained by the fact that pain-related structural changes had already occurred in both groups before imaging, leaving no detectable difference between them. Further longitudinal and mechanistic studies are needed to validate these findings and clarify whether amygdala enlargement represents a pre-existing vulnerability factor, an early marker of subclinical pain, or both."

Reviewer #4 (Remarks to the Author):

This study examines the relationships between neuroticism, polygenetic risk scores and amygdala grey matter and chronic pain onset and recovery over time. The authors identify positive associations between these variables, concluding that neuroticism and genetic risk factors influence the onset of and recovery from chronic pain in important ways, potentially through the amygdala grey matter volume as a neurobiological mechanism, and suggest these factors may even serve as biomarkers for CPM prevention and management. The use of a large dataset and within-study replications on independent subsets are major strengths of the study, and I appreciate the efforts put into testing the robustness of the findings. However, the small effect sizes and lack of critical discussion of the concrete meaning of these findings leave me unconvinced of the clinical relevance of these results.

Major concerns:
While the findings seem robust, the effect sizes for the mediation effects seem extremely small (0.000086-0.00086), diminishing the clinical relevance of these effects. The authors describe this as moderate/modest while in my opinion it would even be a bit of an overstatement to call them small (in my experience, a mediation effect of ~0.01 is generally already considered small). A critical discussion of the meaning of these effects in concrete terms would be needed to evaluate the clinical value of the results.

Response: We fully agree with the reviewer and have revised the Discussion to more accurately characterize the mediation effects as small. We acknowledge that both neuroticism and chronic pain are complex polygenic traits, in which many variants contribute individually small effects. Thus, while statistically significant, these effects are limited in magnitude and should not be overinterpreted in terms of clinical impact. We now frame neuroticism as one contributing factor within a multifactorial process.

The following text was added: "Our mediation analysis revealed that neuroticism mediates the relationship between CMP polygenic risk scores (PRS) and CMP onset, suggesting a potential psychological mechanism through which genetic predisposition may contribute to chronic pain outcomes. However, the observed effect sizes were small and should not be overinterpreted in terms of clinical impact. The indirect effects attributable to neuroticism (Dataset 1: $\beta = 5.64\times10^{-4}$; Dataset 2: $\beta = 3.34\times10^{-4}$) were substantially smaller than their

corresponding total effects (Dataset 1: $\beta = 1.68\times10^{-2}$; Dataset 2: $\beta = 3.14\times10^{-2}$), indicating that neuroticism accounts for only a minor proportion of the overall genetic association with CMP onset. This pattern is further reflected in the logistic regression models, where odds ratios for neuroticism score (OR range: 1.025–1.126; Table 2) and neuroticism PRS (OR range: 1.035–1.093; Table 3) were modest and must be contextualized against baseline pain incidence rather than interpreted as deterministic predictors. Given a baseline CMP incidence of approximately 19.5% in our onset cohort (1,830 of 9,382 pain-free participants; Table 1), an OR of 1.05 translates to an absolute risk increase of roughly one percentage point per neuroticism unit, small at the individual level but potentially meaningful when aggregated across large populations.

These modest effect sizes are nonetheless consistent with prior literature [58–60], which has similarly reported small but significant associations between neuroticism and pain perception, supporting its role as a psychological vulnerability factor influencing pain. Direct comparisons across studies remain limited by differences in outcome definitions and reporting metrics. Small effects are also not unexpected given that both neuroticism and chronic pain are highly polygenic traits, with PRS typically explaining only a modest proportion of phenotypic variance.

A notable distinction emerged between onset and recovery outcomes. While neuroticism significantly mediated the pathway from genetic risk to CMP onset, no significant mediation effect was observed for recovery. This asymmetry suggests that neuroticism primarily acts as a vulnerability factor amplifying genetic susceptibility in pain-free individuals, whereas recovery is likely governed by a distinct and more complex interplay of biological, psychological, and environmental factors. These diverse influences also help explain the considerable individual variability in pain trajectories more broadly. Neuroticism should therefore be understood as one component within a broader multifactorial risk profile. Our findings do not constitute standalone clinical predictors but rather reflect a complex framework in which many factors contribute to CMP onset and recovery. Further research is needed to identify additional mediating pathways and clarify the mechanisms linking genetic risk to CMP outcomes across different stages of pain trajectory."

On a similar note, the authors do not discuss effect sizes for their other analyses, therefore their clinical relevance is not clear. For example, most of the odds ratios for the logistical regressions also seem rather small (1.025-1.093), but it is not explained how we can interpret these findings. For example, I think with an OR of 1.05, the risk for developing chronic pain is 5% higher when neuroticism is x points higher (compared to lower), but it matters a lot if the baseline risk is 0.1% (add 5% and it is 0.005% higher for people with higher neuroticism) or 20% (add 5% and it is 1% higher). This is especially important when these metrics are presented as potential risk factors and/or biomarkers to be used in clinical practice.

Response: We have clarified how to interpret the small odds ratios in the Discussion, adding: "This pattern is further reflected in the logistic regression models, where odds ratios for neuroticism score (OR range: 1.025–1.126; Table 2) and neuroticism PRS (OR

range: 1.035–1.093; Table 3) were modest and must be contextualized against baseline pain incidence rather than interpreted as deterministic predictors. Given a baseline CMP incidence of approximately 19.5% in our onset cohort (1,830 of 9,382 pain-free participants; Table 1), an OR of 1.05 translates to an absolute risk increase of roughly one percentage point per neuroticism unit, small at the individual level but potentially meaningful when aggregated across large populations."

The authors lean into the idea of longitudinal analyses and pain trajectories to aid with causal interpretations. While the time component does add to the relevance of the study, they only use two time points in each analysis and there is little critical discussion of the other major challenge in determining causality, namely the potential for confounding effects. With many years in between measurements, it seems plausible many other factors may have contributed (much more?) to the development and/or recovery of chronic pain. And there may have been other factors that contribute to both neuroticism and chronic pain, but were not included in the analysis.

Response: We have expanded the Limitations section to address unmeasured confounding variables that limit causal conclusions. The following was added, "While our study used multiple timepoints across different datasets, each cohort in our study used two assessment timepoints (a baseline and a single follow-up), so within-person pain trajectories between each timepoint could not be characterized. Despite adjustment for multiple covariates (Table S2), unmeasured variables such as early life adversity, sleep, and socioeconomic stress over the longitudinal study could have contributed to pain outcomes. Thus, causal inference remains limited."

Other concerns:
In the results (line 323-324) it is reported that the relationship between neuroticism and CPM persistence is replicated in both validation datasets, however, in Table 2 the results for dataset C seem to be non-significant?

Response: We thank the reviewer for pointing this out. We have corrected the Results section to accurately reflect that Dataset C did not show a significant association. The following text was added under the Associations Between Neuroticism Score and CMP Onset/Recovery section of the Results: "These significant associations were replicated in the first validation cohort (Dataset B: $p = 0.002$) but were not significant in the second validation cohort (Dataset C: $p = 0.949$). Results for all cohorts can be found in Table 2 and Table S7."

While large and rich datasets like the UK Biobank offer many strengths and advantages, one potential pitfall is the risk of decisions on which data to use and how being influenced by prior knowledge of the data, which makes careful consideration and decision making prior to data analysis (ideally before seeing the data) even more important. Which makes me wonder, were the analyses preregistered prior to analysis? Were any analyses added at a later stage? This would be good to mention in the manuscript for transparency.

Response: We have clarified in the Methods section that the analyses were not preregistered and added a statement to improve transparency: "We formulated our key hypotheses based on prior evidence linking neuroticism, CMP, and amygdala-mediated pain regulation, as outlined in the Introduction, and tested them using the UK Biobank dataset. No major analyses were added subsequently, except those conducted in response to reviewer comments. The study was not publicly preregistered."

The authors mention that analyses were restricted to people of European ancestry and acknowledge this in the limitations of the study. This is essential context for the study results, which would be important to mention in the title or else in the abstract.

Response: We agree and have added this important limitation to the Abstract Methods: "Analyses were restricted to individuals of European ancestry."

The exclusion criteria are quite strict. On one hand, this can help reduce the impact of potential confounding factors. On the other hand, this makes generalization to the general population difficult, especially as chronic pain is often comorbid with other mental and physical health conditions. Many people also experience disability due to chronic pain, does the exclusion criteria relating to disability (line 138) mean they were also excluded from the study? If so, you would get an even more skewed representation of the population of people experiencing chronic pain. These issues would be good to reflect on in the discussion.

Response: We have clarified in the Methods that participants reporting long-term disability or related conditions were excluded. We also added how this may limit generalizability in the Limitations section: "Our study excluded participants with anxiety, depression, long-term disability, and related conditions to isolate neuroticism-specific effects and reduce confounding factors. However, we acknowledge that these are prevalent comorbidities within populations experiencing chronic pain, and their exclusion may have constrained the clinical generalizability of our findings to the broader chronic pain population. Additionally, our exclusion criteria could introduce selection bias toward milder clinical presentations of chronic pain, as we omitted individuals with long-term disability, which can be a manifestation of extreme, disabling pain. This can result in individuals with more severe pain being underrepresented."
