# Supplementary material for: The roles of neuroticism, genetic susceptibility, and amygdala structure in chronic musculoskeletal pain onset and recovery
Source: Commun Med (Lond). 2026 Jul 6;6:567. doi: 10.1038/s43856-026-01754-9 (PMC13719999; doi:10.1038/s43856-026-01754-9)
Supplement: Supplementary file 2 [file 43856_2026_1754_MOESM2_ESM.pdf]

Supplementary materials

The roles of neuroticism, genetic susceptibility, and amygdala structure in chronic musculoskeletal pain onset and recovery

M. Cannistra[1], Y. Liu[1], V. Sacca[1], S. Hodges[1], L. Pham[1], P. Veltri[2], P. H. Lee[1,3] , J. Kong[1]

[1]Department of Psychiatry, Massachusetts General Hospital and Harvard Medical School, Charlestown, MA 02129, USA

[2] Department of Computer, Modeling, Electronics and System Engineering, University of Calabria, Cosenza, Italy

[3] Psychiatric and Neurodevelopmental Genetics Unit, Center for Genomic Medicine, Massachusetts General Hospital, MA, USA

Table of Contents

SUPPLEMENTARY TABLES 3

Table S1 Physiological and neurological disorders defined by an ICD-9 or ICD-10 diagnosis used for exclusion criteria 4

Table S2 The measurement and definition of the variables used in the analyses 5

Table S3 Characteristics of study participants included in the CMP onset analysis (Validation Cohorts) 6

Table S4 Characteristics of study participants included in the CMP recovery analysis (Validation Cohorts) 7

Table S5 Number of participants included in the different datasets used for the analyses 8

Table S6. Logistic regression results for the impact of neuroticism on CMP onset (extended results with all predictors). 9

Table S7. Logistic regression results for the impact of neuroticism on CMP recovery (extended results with all predictors). 10

Table S8. Logistic regression results for the association between the CMP polygenic risk score (PRS) and CMP onset/recovery (extended results with all the predictors). 11

Table S9. Logistic regression results for the association between the neuroticism polygenic risk score (PRS) and CMP onset/recovery (extended results with all the predictors). 12

SUPPLEMENTARY FIGURES 13

Figure S1. Flowchart of the dataset construction for Dataset 1 (Discovery cohort, TP 1 – TP 3) of the chronic musculoskeletal pain (CMP) onset analysis. .... Error! Bookmark not defined.

Figure S2. Flowchart of the dataset construction for Dataset A (Discovery cohort, TP 1 – TP 3) of the chronic musculoskeletal pain (CMP) recovery analysis. .... Error! Bookmark not defined.

Figure S3. Flowchart of the dataset construction for the validation cohorts in the chronic musculoskeletal pain (CMP) onset analysis. .... Error! Bookmark not defined.

Figure S4. Flowchart of the dataset construction for the validation cohorts in the chronic musculoskeletal pain (CMP) recovery analysis. .... Error! Bookmark not defined.

Figure S5. Flowchart of the GWAS cohort construction. Both GWAS were conducted only on participants with European ancestry .... Error! Bookmark not defined.

Figure S6. Results of the mediation analysis using the chronic musculoskeletal pain (CMP) recovery Datasets A-B. .... Error! Bookmark not defined.

SUPPLEMENTARY TABLES

Legends

Table S1 Physiological and neurological disorders defined by an ICD-9 or ICD-10 diagnosis used for exclusion criteria.

Table S2 The measurement and definition of the variables used in the analyses.

Table S3 Characteristics of study participants included in the CMP onset analysis (Validation Cohorts).

Table S4 Characteristics of study participants included in the CMP recovery analysis (Validation Cohorts).

Table S5 Number of participants included in the different datasets used for the analyses.

Table S6 Logistic regression results for the impact of neuroticism on CMP onset (extended results with all predictors).

Table S7 Logistic regression results for the impact of neuroticism on CMP recovery (extended results with all predictors).

Table S8. Logistic regression results for the association between the CMP polygenic risk score (PRS) and CMP onset/recovery (extended results with all predictors).

Table S9. Logistic regression results for the association between the neuroticism polygenic risk score (PRS) and CMP onset/recovery (extended results with all predictors).

Table S1 Physiological and neurological disorders defined by an ICD-9 or ICD-10 diagnosis used for exclusion criteria.

Diagnosis	UK Biobank field code	Disorder
ICD-10	41270	Tuberculosis + Acute poliomyelitis
ICD-10	41270	Benign neoplasms
ICD-10	41270	Organic, including symptomatic, mental disorders
ICD-10	41270	Schizophrenia, schizotypal, and delusional disorders
ICD-10	41270	Manic episode + Bipolar affective disorder
ICD-10	41270	Obsessive-compulsive disorder
ICD-10	41270	Depressive episode
ICD-10	41270	Recurrent depressive disorder
ICD-10	41270	Phobic anxiety disorders
ICD-10	41270	Other anxiety disorders
ICD-10	41270	Reaction to severe stress and adjustment disorders
ICD-10	41270	Other neurotic disorders
ICD-10	41270	Eating disorders
ICD-10	41270	Mental disorder, not otherwise specified
ICD-10	41270	Mental retardation
ICD-10	41270	Disorders of psychological development
ICD-10	41270	Diseases of the nervous system
ICD-10	41270	Cerebral infarction
ICD-10	41270	Stroke, not specified as hemorrhage or infarction
ICD-10	41270	Injuries to the head
ICD-10	41270	Spina bifida
ICD-9	41271	Benign neoplasm of lymph nodes
ICD-9	41271	Anxiety
ICD-9	41271	Cellulitis and abscess of face
ICD-9	41271	Benign neoplasm of lymph nodes
ICD-9	41271	Fracture of skull
ICD-9	41271	Fracture of neck
ICD-9	41271	Congenital anomalies - Nervous system

Table S2 The measurement and definition of the variables used in the analyses.

Variables	UK Biobank Field Code	Description
Gender	31	0: Female, 1: Male
Age	21003	Age in years
Income	738	"What is the average total income before tax received by your HOUSEHOLD?"<br>Annual / Monthly / Weekly<br>1. less than £18,000 / less than £1,500 / less than £346<br>2. £18,000 to £29,999 / £1,500 to £2,499 / £346 to £576<br>3. £30,000 to £51,999 / £2,500 to £4,333 / £577 to £999<br>4. £52,000 to £100,000 / £4,334 to £8,333 / £1000 to £1,923<br>5. greater than £100,000 / greater than £8,333 / greater than £1,923
Smoking	20116	Smoking status:<br>1. Never<br>2. Previous<br>3. Current<br>4. Prefer not to answer
Physical Activity	22040	Summed MET minutes per week for all activity
BMI	23104	Body Mass Index
Townsend Deprivation Index	22189	Townsend deprivation index calculated immediately prior to participant joining UK Biobank. Based on the preceding national census output areas. Each participant is assigned a score corresponding to the output area in which their postcode is located.
Education Level	6138	"Which of the following qualifications do you have? (You can select more than one)"<br>1. College or University degree<br>2. A levels/AS levels or equivalent<br>3. O levels/GCSEs or equivalent<br>4. CSEs or equivalent<br>5. NVQ or HND or HNC or equivalent<br>6. Other professional qualifications (e.g. nursing, teaching)<br>7. None of the above<br>8. Prefer not to answer
Chronic Musculoskeletal Pain	3404, 3571, 3414, 3773	Chronic musculoskeletal pain is defined as pain that persists or recurs for more than three months, as specified in fields 3404, 3571, 3414, and 3773. The relative questions include: (i) "Have you had back pains for more than three months?", (ii) "Have you had hip pains for more than three months?", (iii) "Have you had knee pains for more than three months?", and (iv) "Have you had neck or shoulder pains for more than three months?". Responses options are 'Yes', 'No', 'Do not know', or 'Prefer not to answer'.
Neuroticism Score	1920, 1930, 1940, 1950, 1960, 1970, 1980, 1990, 2000, 2010, 2020, 2030	This is an externally derived summary score of neuroticisms, based on 12 neurotic behavior domains as reported from fields 1920, 1930, 1940, 1950, 1960, 1970, 1980, 1990, 2000, 2010, 2020 and 2030. The relative questions are:<br>"Does your mood often go up and down?"<br>"Do you ever feel 'just miserable' for no reason?"<br>"Are you an irritable person?"<br>"Are your feelings easily hurt?"<br>"Do you often feel 'fed-up'?"<br>"Would you call yourself a nervous person?"<br>"Are you a worrier?"<br>"Would you call yourself tense or 'highly strung'?"<br>"Do you worry too long after an embarrassing experience?"<br>"Do you suffer from 'nerves'?"<br>"Do you often feel lonely?"<br>Are you often troubled by feelings of guilt?"<br>Possible responses are: 'Yes', 'No', 'Do not know', 'Prefer not to answer'.
Worst Pain Intensity, Total Pain Intensity, Number of pain sites	120025, 120026, 120028, 120029	These ratings were derived from fields 120025, 120026, 120028, and 120029 of the web-based pain questionnaires, which included the following questions: In the last 3 months have you experienced pain or discomfort in your neck or shoulders (Back/ Hip/Knee)? Thinking about the last 24 hours, how would you rate your neck and shoulder (Back/Hip/Knee) pain on a 0-10 scale, where 0 is 'no pain' and 10 is 'pain as bad as it could be'?

Table S3 Characteristics of study participants included in the CMP onset analysis (Validation Cohorts).

Variable	Full Sample	Steady Pain-Free	CMP Onset
**Dataset 2 (TP 1 – PQ, Validation Cohort 1)**			
Number of Participants	18,740	12,764	5,976
Age (years)	55.00 ± 7.76	54.87 ± 7.82	55.26 ± 7.63
Sex (N. Female)	9,780 (52.18%)	6,497 (50.90%)	3,283 (54.93%)
BMI (Kg/m^2)	25.93 ± 3.94	25.74 ± 3.84	26.33 ± 4.13
Smoking			
Never	11,588 (61.83%)	8,066 (63.19%)	3,522 (58.93%)
Previous	5,995 (31.99%)	3,923 (30.73%)	2,072 (34.67%)
Current	1,157 (6.17%)	775 (6.07%)	382 (6.39%)
Income			
Less than 18,000£	1,734 (9.25%)	1,144 (8.96%)	590 (9.87%)
18,000 to 30,999£	3,778 (20.16%)	2,546 (19.94%)	1,232 (20.61%)
31,000 to 51,999£	5,358 (28.59%)	3,662 (28.69%)	1,696 (28.38%)
52,000 to 100,000£	5,700 (30.41%)	3,899 (30.54%)	1,801 (30.13%)
Greater than 100,000£	2,170 (11.57%)	1,513 (11.85%)	657 (10.99%)
Physical Activity (hours/week)	41.15 ± 38.22	41.03 ± 38.13	41.40 ± 38.41
Townsend Deprivation Index	-1.78 ± 2.75	-1.77 ± 2.77	-1.79 ± 2.70
Higher Education (college/university degree)	9,996 (53.34%)	6,929 (54.28%)	3,067 (51.32%)
Intermediate Education (A/AS levels or equivalent, O levels/GCSEs or equivalent)	5,853 (31.23%)	3,931 (30.79%)	1,922 (32.16%)
Low Education (none of the above)	2,891 (15.42%)	1,904 (14.91%)	987 (16.51%)
Neuroticism Score	2.95 ± 2.79	2.85 ± 2.77	3.17 ± 2.81
Follow-Up Time (years)	10.01 ± 0.85	10.01 ± 0.86	10.01 ± 0.85
**Dataset 3 (TP 3 – TP 4, Validation Cohort 2)**			
Number of Participants	1,018	864	154
Age (years)	61.11 ± 7.48	61.04 ± 7.17	61.46 ± 7.80
Sex (N. Female)	520 (51.08%)	451 (52.19%)	69 (44.80%)
BMI (Kg/m^2)	25.54 ± 3.65	25.38 ± 3.61	26.42 ± 3.76
Smoking			
Never	697 (68.46%)	596 (68.98%)	101 (65.58%)
Previous	278 (27.30%)	236 (27.31%)	42 (27.27%)
Current	43 (4.22%)	32 (3.70%)	11 (7.14%)
Income			
Less than 18,000£	94 (9.23%)	81 (9.37%)	13 (8.44%)
18,000 to 30,999£	211 (20.72%)	184 (21.29%)	27 (17.53%)
31,000 to 51,999£	309 (30.35%)	259 (29.97%)	50 (32.46%)
52,000 to 100,000£	283 (27.79%)	238 (27.54%)	45 (29.22%)
Greater than 100,000£	121 (11.88%)	102 (11.80%)	19 (12.33%)
Physical Activity (hours/week)	51.18 ± 40.21	50.92 ± 40.96	52.69 ± 35.82
Townsend Deprivation Index	-2.04 ± 2.55	-1.99 ± 2.59	-2.29 ± 2.27
Higher Education (college/university degree)	566 (55.59%)	484 (56.01%)	82 (53.24%)
Intermediate Education (A/AS levels or equivalent, O levels/GCSEs or equivalent)	286 (28.09%)	248 (28.70%)	38 (24.67%)
Low Education (none of the above)	166 (16.30%)	132 (15.27%)	34 (22.07%)
Neuroticism Score	2.47 ± 2.68	2.36 ± 2.56	3.12 ± 3.17
Follow-Up Time (years)	2.99 ± 1.35	2.99 ± 1.35	3.00 ± 1.36

Abbreviations: TP = Timepoint; CMP = Chronic Musculoskeletal Pain; PQ = Pain Questionnaire. The Townsend Deprivation Index is a measure of socioeconomic deprivation.

Table S4 Characteristics of study participants included in the CMP recovery analysis (Validation Cohorts).

Variable	Full Sample	Recovered from CMP	Persistent CMP
**Dataset B (TP 1 – PQ, Validation Cohort 1)**			
Number of Participants	7,217	3,265	3,952
Age (years)	55.31 ± 7.72	54.93 ± 7.78	55.62 ± 7.66
Sex (N. Female)	3,726 (51.62%)	1,583 (48.48%)	2,143 (54.22%)
BMI (Kg/m^2)	26.73 ± 4.24	26.31 ± 4.05	27.09 ± 4.37
Smoking			
Never	4,071 (56.40%)	1,914 (58.62%)	2,157 (54.57%)
Previous	2,558 (35.44%)	1,095 (33.53%)	1,463 (37.01%)
Current	588 (8.14%)	256 (7.84%)	332 (8.40%)
Income			
Less than 18,000£	723 (10.01%)	300 (9.18%)	423 (10.70%)
18,000 to 30,999£	1,542 (21.36%)	674 (20.64%)	868 (21.96%)
31,000 to 51,999£	2,082 (28.84%)	935 (28.63%)	1,147 (29.02%)
52,000 to 100,000£	2,114 (29.29%)	976 (29.89%)	1,138 (28.79%)
Greater than 100,000£	756 (10.47%)	380 (11.63%)	376 (9.51%)
Physical Activity (hours/week)	42.84 ± 39.98	42.42 ± 39.79	43.19 ± 40.14
Townsend Deprivation Index	-1.84 ± 2.73	-1.77 ± 2.76	-1.90 ± 2.71
High Education (college or university degree)	3,424 (47.44%)	1,578 (48.33%)	1,846 (46.71%)
Intermediate Education (A/AS levels or equivalent, O levels/GCSEs or equivalent)	2,379 (32.96%)	1,059 (32.43%)	1,320 (33.40%)
Low Education (none of the above)	1,414 (19.59%)	628 (19.23%)	786 (19.88%)
Neuroticism Score	3.69 ± 3.00	3.58 ± 3.02	3.79 ± 2.98
Follow-Up Time (years)	10.03 ± 0.85	10.02 ± 0.85	10.04 ± 0.85
**Dataset C (TP 3 – TP 4, Validation Cohort 2)**			
Number of Participants	332	140	192
Age (years)	61.30 ± 7.60	60.45 ± 7.41	61.91 ± 7.69
Sex (N. Female)	168 (50.60%)	67 (47.85%)	101 (52.60%)
BMI (Kg/m^2)	26.64 ± 4.18	26.39 ± 4.48	26.82 ± 3.94
Smoking			
Never	213 (64.15%)	90 (64.28%)	123 (64.06%)
Previous	111 (33.43%)	46 (32.85%)	65 (33.85%)
Current	8 (2.40%)	4 (2.85%)	4 (2.08%)
Income			
Less than 18,000£	25 (7.53%)	10 (7.14%)	15 (7.81%)
18,000 to 30,999£	93 (28.01%)	33 (23.57%)	60 (31.25%)
31,000 to 51,999£	99 (29.81%)	44 (31.42%)	55 (28.64%)
52,000 to 100,000£	82 (24.69%)	38 (27.14%)	44 (22.91%)
Greater than 100,000£	33 (9.93%)	15 (10.71%)	18 (9.37%)
Physical Activity (hours/week)	52.89 ± 43.99	48.33 ± 38.43	56.22 ± 47.48
Townsend Deprivation Index	-2.26 ± 2.38	-2.21 ± 2.43	-2.30 ± 2.35
High Education (college or university degree)	147 (44.27%)	65 (46.42%)	82 (42.70%)
Intermediate Education (A/AS levels or equivalent, O levels/GCSEs or equivalent)	122 (36.74%)	56 (40.00%)	66 (34.37%)
Low Education (none of the above)	63 (18.97%)	19 (13.57%)	44 (22.91%)
Neuroticism Score	3.46 ± 3.05	3.51 ± 3.16	3.42 ± 2.97
Follow-Up Time (years)	2.97 ± 1.37	2.90 ± 1.30	3.03 ± 1.42

Abbreviations: TP = Timepoint; CMP = Chronic Musculoskeletal Pain; PQ= Pain Questionnaire. The Townsend Deprivation Index is a measure of socioeconomic deprivation.

Table S5 Number of participants included in the different datasets used for the analyses.

Cohort	Analysis	Full Sample	Steady Pain-Free	CMP Onset
**CMP Onset Dataset**				
Dataset 1 (TP 1 – TP 3)	Primary analysis (neuroticism – CMP onset)	9,382	7,552	1,830
	Subset with genetic data	9.221	7,414	1,807
Dataset 2 (TP 1 – PQ)	Primary analysis (neuroticism – CMP onset)	18,740	12,764	5,976
	Subset with genetic data	18,407	12,527	5,880
Dataset 3 (TP 3 – TP 4)	Primary analysis (neuroticism – CMP onset)	1,018	864	154
	Subset with imaging data	847	722	125
**CMP Recovery Dataset**		**Full Sample**	**CMP Recovery**	**Persistent CMP**
Dataset A (TP 1 – TP 3)	Primary analysis (neuroticism – CMP recovery)	3,460	1,863	1,597
	Subset with genetic data	3,385	1,825	1,560
Dataset B (TP 1 – PQ)	Primary analysis (neuroticism – CMP recovery)	7,217	3,265	3,952
	Subset with genetic data	7,104	3,206	3,898
Dataset C (TP 3 – TP 4)	Primary analysis (neuroticism – CMP recovery)	332	140	192
	Subset with imaging data	293	123	170

Abbreviations: TP = Time Point; CMP = Chronic Musculoskeletal Pain; PQ = Pain Questionnaire.

Table S6. Logistic regression results for the impact of neuroticism on CMP onset (extended results with all predictors).

Dataset	p-value	Odds Ratio [95% CI]
**Dataset 1 (TP 1 – TP 3, Discovery Cohort)**		
Age	0.831	0.999 [0.991 – 1.006]
Sex	0.030	0.889 [0.799 – 0.989]
BMI	5.07 e-15	1.056 [1.042 – 1.071]
Smoking	1.87 e-5	1.205 [1.106 – 1.313]
Income	0.147	0.962 [0.914 – 1.013]
Physical Activity	0.012	1.000028 [1.000006 – 1.000048]
Townsend Deprivation Index	0.598	0.994 [0.975 – 1.014]
Education	0.037	0.926 [0.862 – 0.995]
Follow-up Time	0.174	1.013 [0.994 – 1.033]
Neuroticism Score	7.92 e-9	1.055 [1.036 – 1.074]
**Dataset 2 (TP 1 – PQ, Validation Cohort 1)**		
Age	0.001	1.007 [1.002 – 1.011]
Sex	6.69 e-10	0.817 [0.767 – 0.871]
BMI	2.02 e-25	1.042 [1.034 – 1.051]
Smoking	1.97 e-5	1.117 [1.061 – 1.176]
Income	0.462	1.011 [0.981 – 1.042]
Physical Activity	0.123	1.00001 [0.999 – 1.00002]
Townsend Deprivation Index	0.416	0.995 [0.983 – 1.006]
Education	0.101	0.963 [0.921 – 1.007]
Follow-up Time	0.849	1.003 [0.967 – 1.040]
Neuroticism Score	1.82 e-13	1.042 [1.031 – 1.054]
**Dataset 3 (TP 3 – TP 4, Validation Cohort 2)**		
Age	0.264	1.014 [0.988 – 1.041]
Sex	0.134	1.320 [0.917 – 1.900]
BMI	0.003	1.072 [1.023 – 1.124]
Smoking	0.361	1.153 [0.848 – 1.569]
Income	0.097	1.155 [0.973 – 1.370]
Physical Activity	0.427	1.00002 [0.999 – 1.0001]
Townsend Deprivation Index	0.155	0.948 [0.881 – 1.020]
Education	0.259	0.874 [0.692 – 1.104]
Follow-up Time	0.675	1.028 [0.902 – 1.171]
Neuroticism Score	0.0001	1.126 [1.058 – 1.199]

Abbreviations: TP = Time Point; CMP = Chronic Musculoskeletal Pain; PQ = Pain Questionnaire. All the models were adjusted for age, gender, BMI, smoking, income, townsend deprivation index, education, physical activity, and follow-up time. All the coefficients were tested using two-sided tests.

Table S7. Logistic regression results for the impact of neuroticism on CMP recovery (extended results with all predictors).

Dataset	p-value	Odds Ratio [95% CI]
**Dataset A (TP 1 – TP 3, Discovery Cohort)**		
Age	0.121	0.992 [0.982 – 1.002]
Sex	0.269	0.925 [0.805 – 1.062]
BMI	4.08 e-9	1.054 [1.035 – 1.073]
Smoking	0.316	0.943 [0.842 – 1.057]
Income	0.614	1.017 [0.950 – 1.089]
Physical Activity	0.003	1.00004 [1.00001 – 1.00007]
Townsend Deprivation Index	0.652	1.006 [0.979 – 1.032]
Education	0.079	0.920 [0.839 – 1.009]
Follow-up Time	0.935	1.001 [0.975 – 1.026]
Neuroticism Score	0.001	1.038 [1.014 – 1.062]
**Dataset B (TP 1 – PQ, Validation Cohort 1)**		
Age	0.009	1.008 [1.002 – 1.015]
Sex	1.75 e-8	0.758 [0.689 – 0.835]
BMI	1.73 e-16	1.049 [1.037 – 1.062]
Smoking	0.001	1.129 [1.049 – 1.216]
Income	0.110	0.963 [0.920 – 1.008]
Physical Activity	0.109	1.00001 [0.999 – 1.00003]
Townsend Deprivation Index	0.002	0.973 [0.956 – 0.990]
Education	0.348	1.031 [0.966 – 1.101]
Follow-up Time	0.304	1.029 [0.974 – 1.088]
Neuroticism Score	0.002	1.025 [1.008 – 1.041]
**Dataset C (TP 3 – TP 4, Validation Cohort 2)**		
Age	0.121	1.026 [0.993 – 1.060]
Sex	0.226	0.753 [0.476 – 1.192]
BMI	0.238	1.033 [0.978 – 1.092]
Smoking	0.787	0.944 [0.621 – 1.434]
Income	0.997	0.999 [0.792 – 1.260]
Physical Activity	0.103	1.00007 [0.999 – 1.0001]
Townsend Deprivation Index	0.904	0.994 [0.903 – 1.093]
Education	0.268	0.834 [0.604 – 1.150]
Follow-up Time	0.314	1.089 [0.921 – 1.288]
Neuroticism Score	0.949	0.997 [0.924 – 1.075]

Abbreviations: TP = Time Point; CMP = Chronic Musculoskeletal Pain; PQ = Pain Questionnaire. All the models were adjusted for age, gender, BMI, smoking, income, townsend deprivation index, education, physical activity, and follow-up time. All the coefficients were tested using two-sided tests.

Table S8. Logistic regression results for the association between the CMP polygenic risk score (PRS) and CMP onset/recovery (extended results with all predictors).

Dataset	p-value	Odds Ratio [95% CI]
**CMP Onset Data**		
**Dataset 1 (TP 1 – TP 3, Discovery Cohort)**		
Age	0.707	0.998 [0.991 – 1.006]
Sex	0.005	0.860 [0.773 – 0.957]
BMI	5.79 e-12	1.050 [1.035 – 1.065]
Smoking	1.91 e-5	1.207 [1.107 – 1.316]
Income	0.093	0.956 [0.908 – 1.007]
Physical Activity	0.036	1.00002 [1.000001 – 1.00004]
Townsend Deprivation Index	0.535	0.993 [0.973 – 1.013]
Education	0.056	0.931 [0.866 – 1.001]
Follow-up Time	0.213	1.012 [0.992 – 1.032]
CMP PRS	3.76 e-11	1.197 [1.135 – 1.262]
PC1	0.171	0.998 [0.996 – 1.0006]
PC2	0.511	0.999 [0.996 – 1.001]
PC3	0.403	0.997 [0.992 – 1.002]
PC4	0.470	1.002 [0.996 – 1.008]
PC5	0.342	1.003 [0.996 – 1.010]
PC6	0.968	1.0004 [0.978 – 1.022]
PC7	0.110	0.989 [0.976 – 1.002]
PC8	0.773	1.002 [0.986 – 1.018]
PC9	0.923	0.999 [0.986 – 1.012]
PC10	0.901	1.001 [0.983 – 1.018]
**Dataset 2 (TP 1 – PQ, Validation Cohort 1)**		
Age	0.025	1.004 [1.0006 – 1.009]
Sex	2.30 e-12	0.795 [0.745 – 0.847]
BMI	3.29 e-20	1.038 [1.030 – 1.046]
Smoking	2.16 e-5	1.118 [1.062 – 1.177]
Income	0.805	1.003 [0.973 – 1.034]
Physical Activity	0.331	1.000007 [0.999 – 1.00002]
Townsend Deprivation Index	0.620	0.997 [0.985 – 1.008]
Education	0.255	0.974 [0.931 – 1.019]
Follow-up Time	0.839	1.003 [0.967 – 1.042]
CMP PRS	2.55 e-15	1.137 [1.101 – 1.173]
PC1	0.036	0.998 [0.998 – 0.999]
PC2	0.678	1.0003 [0.998 – 1.002]
PC3	0.440	0.998 [0.995 – 1.001]
PC4	0.613	0.999 [0.995 – 1.002]
PC5	0.332	0.997 [0.993 – 1.002]
PC6	0.756	0.998 [0.990 – 1.007]
PC7	0.924	0.999 [0.992 – 1.006]
PC8	0.618	0.998 [0.990 – 1.005]
PC9	0.218	1.004 [0.997 – 1.011]
PC10	0.713	1.001 [0.991 – 1.012]
**CMP Recovery Data**		
**Dataset A (TP 1 – TP 3, Discovery Cohort)**		
Age	0.046	0.990 [0.980 – 0.999]
Sex	0.254	0.922 [0.801 – 1.060]
BMI	2.73 e-7	1.048 [1.029 – 1.067]
Smoking	0.239	0.932 [0.830 – 1.047]
Income	0.910	1.003 [0.936 – 1.076]
Physical Activity	0.019	1.00003 [1.000006 – 1.00006]
Townsend Deprivation Index	0.747	1.004 [0.977 – 1.032]
Education	0.073	0.917 [0.834 – 1.008]
Follow-up Time	0.905	0.998 [0.972 – 1.024]
CMP PRS	4.78 e-6	1.178 [1.098 – 1.264]
PC1	0.994	0.999 [0.997 – 1.002]
PC2	0.727	1.0006 [0.997 – 1.004]
PC3	0.795	1.0007 [0.994 – 1.006]
PC4	0.379	0.996 [0.988 – 1.004]
PC5	0.019	1.011 [1.001 – 1.020]
PC6	0.375	0.987 [0.961 – 1.015]
PC7	0.907	1.001 [0.983 – 1.018]
PC8	0.710	1.003 [0.983 – 1.024]
PC9	0.018	0.980 [0.963 – 0.996]
PC10	0.490	1.007 [0.985 – 1.030]
**Dataset B (TP 1 – PQ, Validation Cohort 1)**		
Age	0.039	1.006 [1.0003 – 1.013]
Sex	5.14 e-9	0.750 [0.681 – 0.826]
BMI	1.08 e-14	1.047 [1.035 – 1.059]
Smoking	0.001	1.126 [1.045 – 1.213]
Income	0.054	0.955 [0.912 – 1.0009]
Physical Activity	0.189	1.00001 [0.999 – 1.00003]
Townsend Deprivation Index	0.009	0.976 [0.959 – 0.994]
Education	0.324	1.033 [0.967 – 1.103]
Follow-up Time	0.502	1.019 [0.963 – 1.078]
CMP PRS	0.010	1.064 [1.014 – 1.117]
PC1	0.221	0.999 [0.997 – 1.0005]
PC2	0.522	1.0008 [0.998 – 1.003]
PC3	0.894	0.999 [0.995 – 1.004]
PC4	0.554	0.998 [0.993 – 1.003]
PC5	0.832	0.999 [0.992 – 1.005]
PC6	0.521	0.996 [0.984 – 1.007]
PC7	0.402	0.995 [0.985 – 1.006]
PC8	0.494	1.003 [0.992 – 1.014]
PC9	0.442	1.004 [0.993 – 1.014]
PC10	0.655	0.996 [0.982 – 1.011]

Abbreviations: TP = Time Point; CMP = Chronic Musculoskeletal Pain; PQ = Pain Questionnaire; PC = Principal Component. All the models were adjusted for age, gender, BMI, smoking, income, townsend deprivation index, education, physical activity, follow-up time, and first 10 PCs. All the coefficients were tested using two-sided tests.

Table S9. Logistic regression results for the association between the neuroticism polygenic risk score (PRS) and CMP onset/recovery (extended results with all predictors).

Dataset	p-value	Odds Ratio [95% CI]
**CMP Onset Data**		
**Dataset 1 (TP 1 – TP 3, Discovery Cohort)**		
Age	0.561	0.997 [0.990 – 1.005]
Sex	0.004	0.858 [0.771 – 0.954]
BMI	8.99 e-14	1.054 [1.040 – 1.069]
Smoking	1.50 e-5	1.210 [1.110 – 1.319]
Income	0.051	0.949 [0.901 – 1.0005]
Physical Activity	0.025	1.00002 [1.000003 – 1.00004]
Townsend Deprivation Index	0.563	0.994 [0.974 – 1.014]
Education	0.026	0.921 [0.857 – 0.990]
Follow-up Time	0.220	1.012 [0.992 – 1.032]
Neuroticism PRS	7.66 e-4	1.093 [1.038 – 1.152]
PC1	0.330	0.998 [0.992 – 1.001]
PC2	0.574	0.999 [0.996 – 1.002]
PC3	0.527	0.998 [0.993 – 1.003]
PC4	0.363	1.002 [0.996 – 1.008]
PC5	0.377	1.003 [0.996 – 1.010]
PC6	0.939	1.0008 [0.978 – 1.023]
PC7	0.126	0.989 [0.977 – 1.002]
PC8	0.749	1.002 [0.986 – 1.018]
PC9	0.936	1.0005 [0.987 – 1.013]
PC10	0.912	1.0009 [0.983 – 1.018]
**Dataset 2 (TP 1 – PQ, Validation Cohort 1)**		
Age	0.045	1.004 [1.00008 – 1.008]
Sex	3.65 e-12	0.796 [0.747 – 0.849]
BMI	2.00 e-23	1.041 [1.033 – 1.049]
Smoking	1.16 e-5	1.121 [1.065 – 1.181]
Income	0.868	1.002 [0.972 – 1.033]
Physical Activity	0.242	1.000008 [0.999 – 1.00002]
Townsend Deprivation Index	0.532	0.996 [0.984 – 1.008]
Education	0.123	0.965 [0.922 – 1.009]
Follow-up Time	0.950	1.001 [0.964 – 1.039]
Neuroticism PRS	0.027	1.035 [1.003 – 1.068]
PC1	0.171	0.999 [0.998 – 1.0002]
PC2	0.431	1.0006 [0.999 – 1.002]
PC3	0.508	0.998 [0.995 – 1.002]
PC4	0.887	0.999 [0.996 – 1.002]
PC5	0.257	0.997 [0.993 – 1.001]
PC6	0.680	0.998 [0.989 – 1.006]
PC7	0.961	0.999 [0.992 – 1.007]
PC8	0.642	0.998 [0.990 – 1.005]
PC9	0.212	1.004 [0.997 – 1.011]
PC10	0.717	1.001 [0.991 – 1.012]
**CMP Recovery Data**		
**Dataset A (TP 1 – TP 3, Discovery Cohort)**		
Age	0.028	0.989 [0.979 – 0.998]
Sex	0.173	0.907 [0.789 – 1.043]
BMI	3.03 e-8	1.052 [1.033 – 1.071]
Smoking	0.307	0.941 [0.838 – 1.056]
Income	0.884	1.005 [0.937 – 1.077]
Physical Activity	0.013	1.00003 [1.000009 – 1.00006]
Townsend Deprivation Index	0.836	1.002 [0.976 – 1.030]
Education	0.031	0.901 [0.820 – 0.991]
Follow-up Time	0.907	0.998 [0.972 – 1.024]
Neuroticism PRS	0.023	1.082 [1.010 – 1.160]
PC1	0.755	1.0004 [0.997 – 1.003]
PC2	0.601	1.0009 [0.997 – 1.004]
PC3	0.725	1.001 [0.995 – 1.006]
PC4	0.449	0.996 [0.988 – 1.005]
PC5	0.270	0.998 [0.977 – 1.019]
PC6	0.365	0.988 [0.962 – 1.014]
PC7	0.770	1.002 [0.985 – 1.020]
PC8	0.706	1.003 [0.983 – 1.024]
PC9	0.230	0.987 [0.964 – 1.010]
PC10	0.419	1.009 [0.986 – 1.031]
**Dataset B (TP 1 – PQ, Validation Cohort 1)**		
Age	0.047	1.006 [1.00008 – 1.013]
Sex	3.86 e-9	0.748 [0.679 – 0.824]
BMI	1.39 e-15	1.048 [1.036 – 1.061]
Smoking	0.001	1.129 [1.047 – 1.216]
Income	0.056	0.955 [0.912 – 1.001]
Physical Activity	0.149	1.00001 [0.999 – 1.00003]
Townsend Deprivation Index	0.010	0.976 [0.959 – 0.994]
Education	0.384	1.029 [0.964 – 1.099]
Follow-up Time	0.489	1.020 [0.964 – 1.079]
Neuroticism PRS	0.083	1.042 [0.994 – 1.093]
PC1	0.295	0.999 [0.997 – 1.0006]
PC2	0.452	1.0009 [0.998 – 1.003]
PC3	0.925	0.999 [0.995 – 1.004]
PC4	0.591	0.998 [0.993 – 1.003]
PC5	0.687	0.998 [0.992 – 1.005]
PC6	0.516	0.996 [0.984 – 1.007]
PC7	0.443	0.995 [0.985 – 1.006]
PC8	0.530	1.003 [0.992 – 1.014]
PC9	0.464	1.003 [0.993 – 1.014]
PC10	0.624	0.996 [0.982 – 1.010]

Abbreviations: TP = Time Point; CMP = Chronic Musculoskeletal Pain; PQ = Pain Questionnaire; PC = Principal Component. All the models were adjusted for age, gender, BMI, smoking, income, townsend deprivation index, education, physical activity, follow-up time, and first 10 PCs. All the coefficients were tested using two-sided tests.

SUPPLEMENTARY FIGURES

Legends.

Figure S1. Flowchart of the dataset construction for Dataset 1 (Discovery cohort, TP 1 – TP 3) of the chronic musculoskeletal pain (CMP) onset analysis. Participants were first filtered to remove those with psychological or neurological disorders identified by ICD-10 or ICD-9 diagnosis, a diagnosis of diabetes, or long-standing illness, disability, or infirmity. Only pain-free participants without acute or chronic pain of any type were then included in the analysis. Among the remaining participants, those who remained steadily pain-free across assessments (Group 1) and those who developed a CMP at the follow-up timepoint (TP 3) were identified. Participants with missing values or uninterpretable answers (e.g., 'do not know' or 'prefer not to answer') for any analysis variable (age, gender, BMI, income, smoking, Townsend deprivation index, physical activity, education, and the 12 variables used to compute the neuroticism score) were excluded. The remaining cohort was used to assess the neuroticism – CMP onset association. A subset of participants with available genetic data was then extracted to examine the CMP polygenic risk score (PRS) – CMP onset association and the neuroticism PRS – neuroticism phenotype association.

Figure S2. Flowchart of the dataset construction for Dataset A (Discovery cohort, TP 1 – TP 3) of the chronic musculoskeletal pain (CMP) recovery analysis. Participants were first filtered to remove those with psychological or neurological disorders identified by ICD-10 or ICD-9 diagnosis, a diagnosis of diabetes, or long-standing illness, disability, or infirmity. Only participants who reported CMP without any other coexisting pain (headaches, facial pain, abdominal pain, general pain) were included in the analysis. Within this cohort, those who recovered from CMP (Group 1) and those with persistent CMP across assessments (Group 2) were identified. Participants with missing values or uninterpretable answers (e.g., 'do not know' or 'prefer not to answer') for any analysis variable (age, gender, BMI, income, smoking, Townsend deprivation index, physical activity, education, and the 12 variables used to build the neuroticism score) were excluded. The remaining cohort was used to assess the neuroticism – CMP recovery association. A subset of participants who also had the genetic data was then extracted to examine the CMP polygenic risk score (PRS) – CMP recovery association and the neuroticism PRS – neuroticism phenotype association.

Figure S3. Flowchart of the dataset construction for the validation cohorts in the chronic musculoskeletal pain (CMP) onset analysis. Participants were first filtered to remove those with psychological or neurological disorders identified by ICD-10 or ICD-9 diagnosis, a diagnosis of diabetes, or long-standing illness, disability, or infirmity. Those without acute or chronic pain were then selected and grouped into those who remained pain-free across assessments and those who developed CMP. Participants with missing values or uninterpretable answers (e.g., 'do not know' or 'prefer not to answer') were then excluded. Dataset 2 leveraged timepoint (TP) 1 as baseline, with follow-up assessments established at the Pain Questionnaire (PQ). Dataset 3 was, instead, built by extracting the healthy controls at TP 3 and assessing their follow-up conditions using TP 4. Any participants already included in other cohorts (i.e., the discovery cohort, Dataset 1), were removed from these validation cohorts. Subsets of participants with available genetic and/or imaging data were extracted from Datasets 2 and 3.

Figure S4. Flowchart of the dataset construction for the validation cohorts in the chronic musculoskeletal pain (CMP) recovery analysis. Participants were first filtered to remove those with psychological or neurological disorders identified by ICD-10 or ICD-9 diagnosis, a diagnosis of diabetes, or long-standing illness, disability, or infirmity. Those who reported at least one CMP site (back, hip, knee, and neck) were included and grouped into those who recovered from CMP and those who reported persistent CMP. Participants with missing values or uninterpretable answers (e.g., 'do not know' or 'prefer not to answer') were then excluded. Dataset B leveraged timepoint (TP) 1 as baseline, with follow-up assessments established at the Pain Questionnaire (PQ). Dataset C was built by extracting the healthy controls at TP 3 and assessing their follow-up conditions using TP 4. Any participants already included in other cohorts (i.e., the discovery cohort, Dataset A) were removed from these validation cohorts. The subset of participants with either genetic and/or imaging data were extracted from Datasets B and C.

Figure S5. Flowchart of the GWAS cohort construction. Both GWAS were conducted only on participants with European ancestry. Quality control procedures were applied to remove participants with sex discrepancy (between self-reported and genetically inferred sex), high missingness, sex chromosome aneuploidy, and relatedness. In the CMP GWAS cohort, pain-free and CMP participants were extracted, excluding those with acute pain or coexisting non-musculoskeletal pain (headaches, general pain, facial pain, and abdominal pain). In the neuroticism GWAS cohort, participants with missing values or uninterpretable answers for any variable used to compute the neuroticism score were excluded.

Figure S6. Results of the mediation analysis using the chronic musculoskeletal pain (CMP) recovery Datasets A-B. Neuroticism served as the mediator of the association between the CMP polygenic risk score (PRS), which was used as the independent variable, and the binary outcome (steady pain-free vs. CMP recovery) in Dataset A (TP 1 – TP 3). These results were not replicated in Dataset B (TP 1 – PQ).

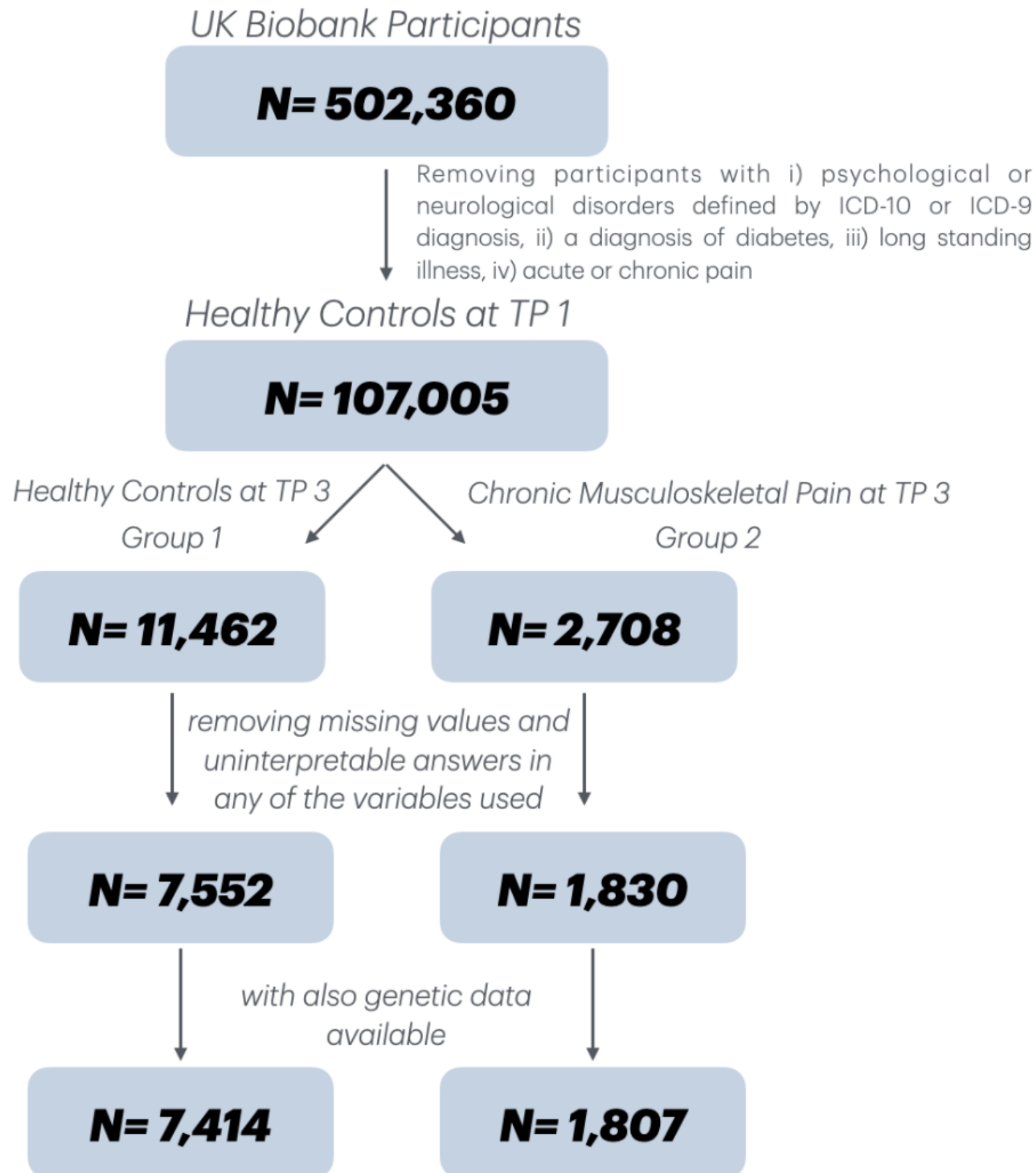

Figure S1. Flowchart of the dataset construction for Dataset 1 (Discovery cohort, TP 1 – TP 3) of the chronic musculoskeletal pain (CMP) onset analysis. Participants were first filtered to remove those with psychological or neurological disorders identified by ICD-10 or ICD-9 diagnosis, a diagnosis of diabetes, or long-standing illness, disability, or infirmity. Only pain-free participants without acute or chronic pain of any type were then included in the analysis. Among the remaining participants, those who remained steadily pain-free across assessments (Group 1) and those who developed a CMP at the follow-up timepoint (TP 3) were identified. Participants with missing values or uninterpretable answers (e.g., 'do not know' or 'prefer not to answer') for any analysis variable (age, gender, BMI, income, smoking, Townsend deprivation index, physical activity, education, and the 12 variables used to compute the neuroticism score) were excluded. The remaining cohort was used to assess the neuroticism – CMP onset association. A subset of participants with available genetic data was then extracted to examine the CMP polygenic risk score (PRS) – CMP onset association and the neuroticism PRS – neuroticism phenotype association.

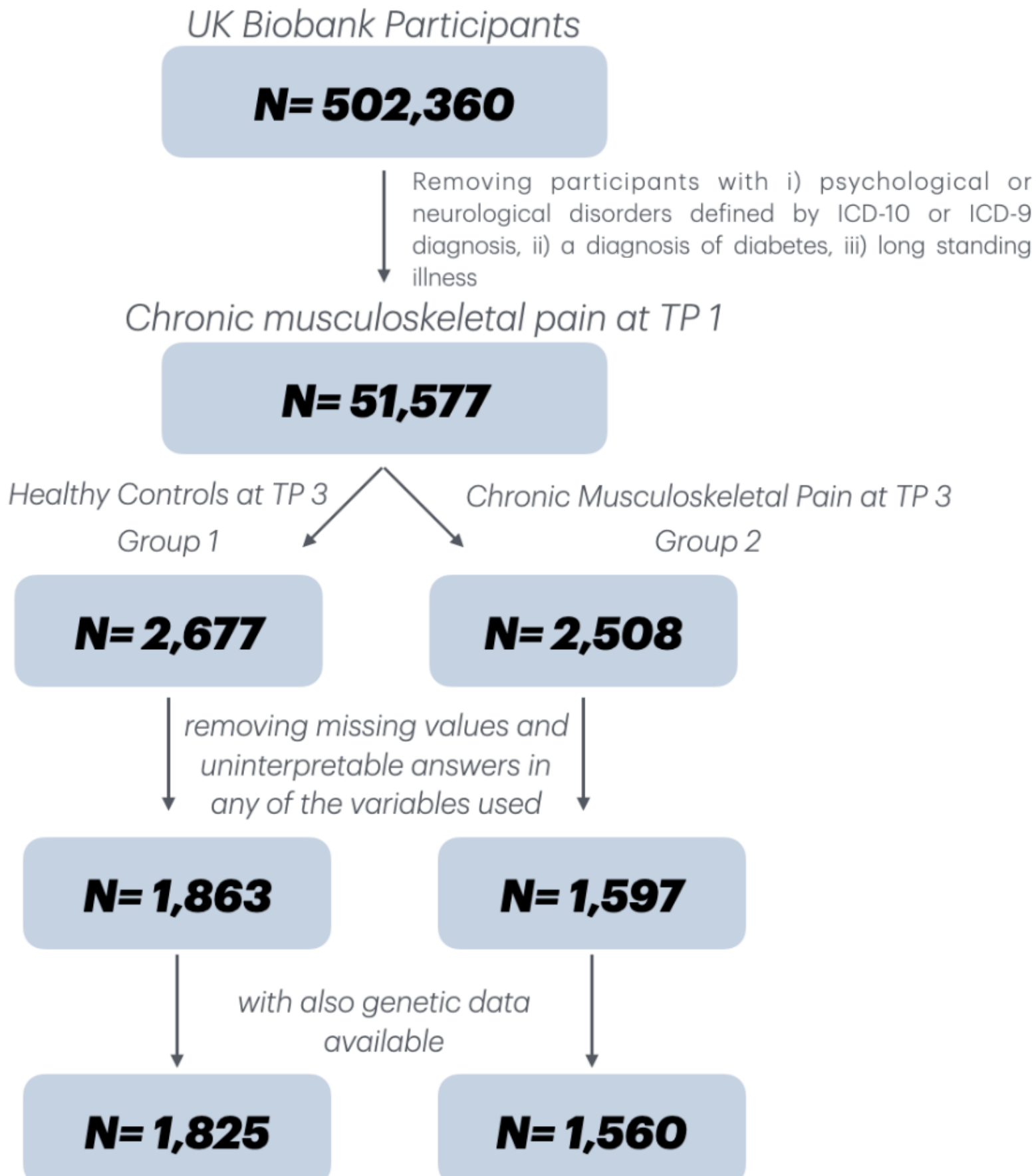

Figure S2. Flowchart of the dataset construction for Dataset A (Discovery cohort, TP 1 – TP 3) of the chronic musculoskeletal pain (CMP) recovery analysis. Participants were first filtered to remove those with psychological or neurological disorders identified by ICD-10 or ICD-9 diagnosis, a diagnosis of diabetes, or long-standing illness, disability, or infirmity. Only participants who reported CMP without any other coexisting pain (headaches, facial pain, abdominal pain, general pain) were included in the analysis. Within this cohort, those who recovered from CMP (Group 1) and those with persistent CMP across assessments (Group 2) were identified. Participants with missing values or uninterpretable answers (e.g., 'do not know' or 'prefer not to answer') for any analysis variable (age, gender, BMI, income, smoking, Townsend deprivation index, physical activity, education, and the 12 variables used to build the neuroticism score) were excluded. The remaining cohort was used to assess the neuroticism – CMP recovery association. A subset of participants who also had the genetic data was then extracted to examine the CMP polygenic risk score (PRS) – CMP recovery association and the neuroticism PRS – neuroticism phenotype association.

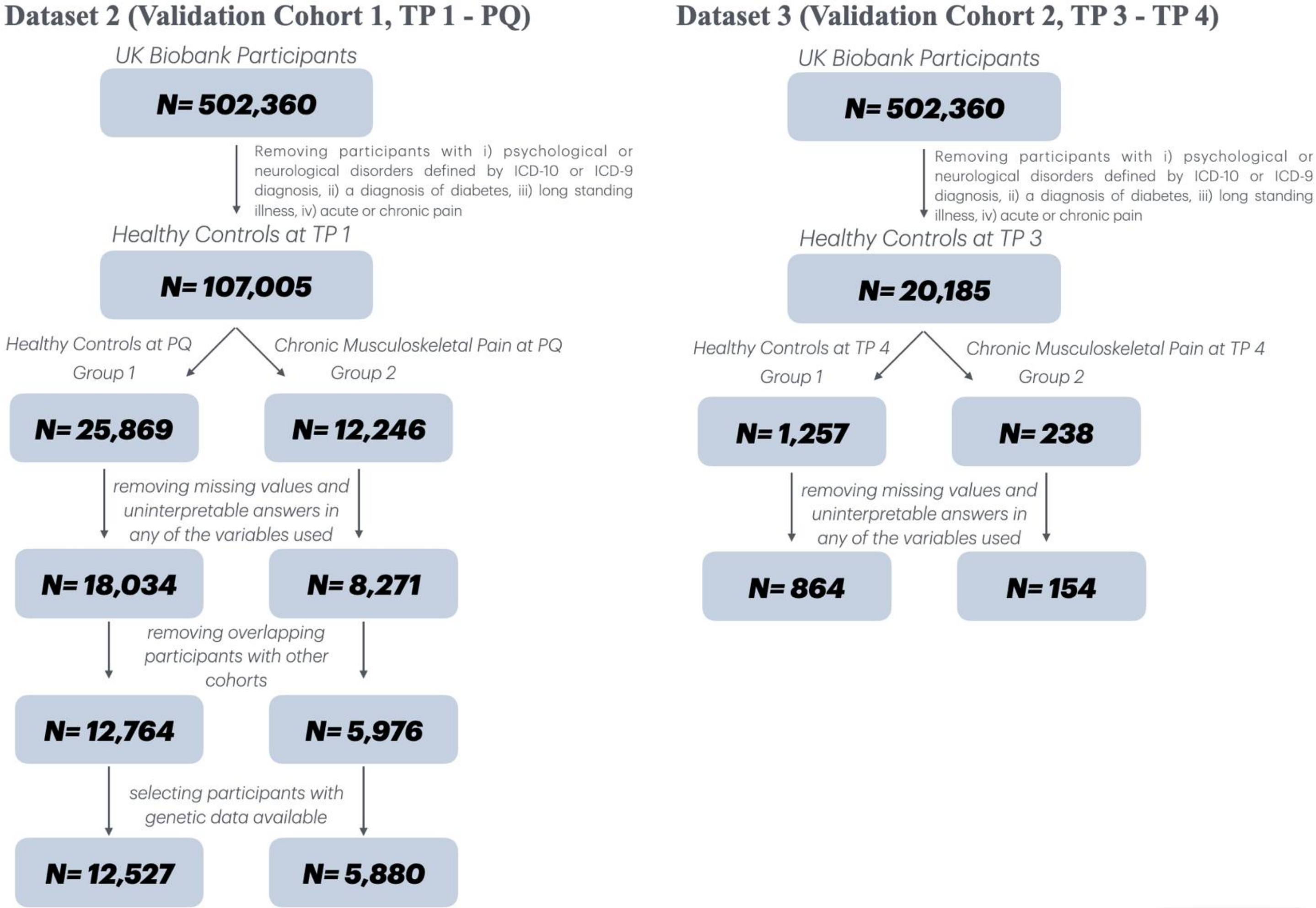

Figure S3. Flowchart of the dataset construction for the validation cohorts in the chronic musculoskeletal pain (CMP) onset analysis. Participants were first filtered to remove those with psychological or neurological disorders identified by ICD-10 or ICD-9 diagnosis, a diagnosis of diabetes, or long-standing illness, disability, or infirmity. Those without acute or chronic pain were then selected and grouped into those who remained pain-free across assessments and those who developed CMP. Participants with missing values or uninterpretable answers (e.g., ‘do not know’ or ‘prefer not to answer’) were then excluded. Dataset 2 leveraged timepoint (TP) 1 as baseline, with follow-up assessments established at the Pain Questionnaire (PQ). Dataset 3 was, instead, built by extracting the healthy controls at TP 3 and assessing their follow-up conditions using TP 4. Any participants already included in other cohorts (i.e., the discovery cohort, Dataset 1), were removed from these validation cohorts. Subsets of participants with available genetic and/or imaging data were extracted from Datasets 2 and 3.

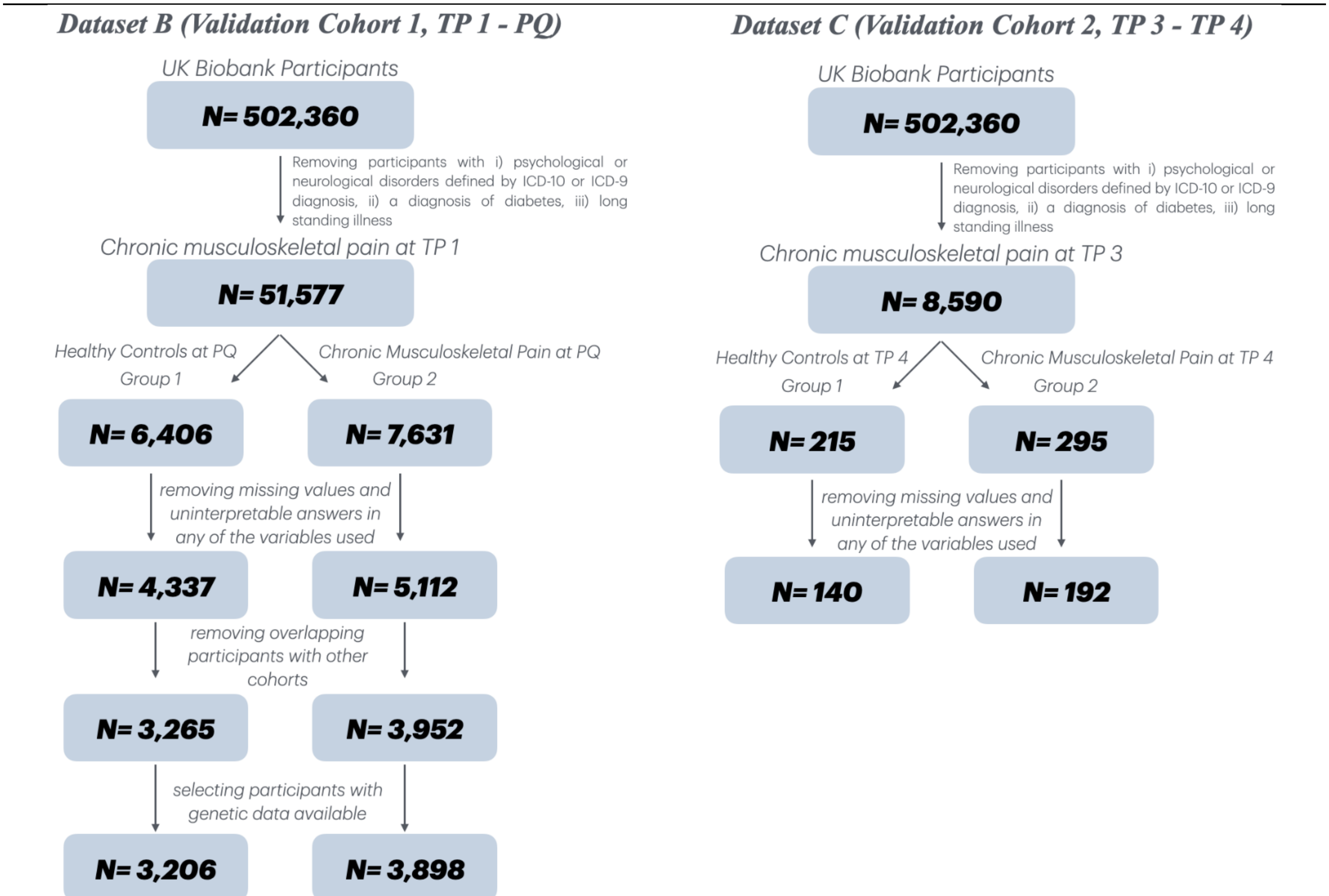

Figure S4. Flowchart of the dataset construction for the validation cohorts in the chronic musculoskeletal pain (CMP) recovery analysis. Participants were first filtered to remove those with psychological or neurological disorders identified by ICD-10 or ICD-9 diagnosis, a diagnosis of diabetes, or long-standing illness, disability, or infirmity. Those who reported at least one CMP site (back, hip, knee, and neck) were included and grouped into those who recovered from CMP and those who reported persistent CMP. Participants with missing values or uninterpretable answers (e.g., 'do not know' or 'prefer not to answer') were then excluded. Dataset B leveraged timepoint (TP) 1 as baseline, with follow-up assessments established at the Pain Questionnaire (PQ). Dataset C was built by extracting the healthy controls at TP 3 and assessing their follow-up conditions using TP 4. Any participants already included in other cohorts (i.e., the discovery cohort, Dataset A) were removed from these validation cohorts. The subset of participants with either genetic and/or imaging data were extracted from Datasets B and C.

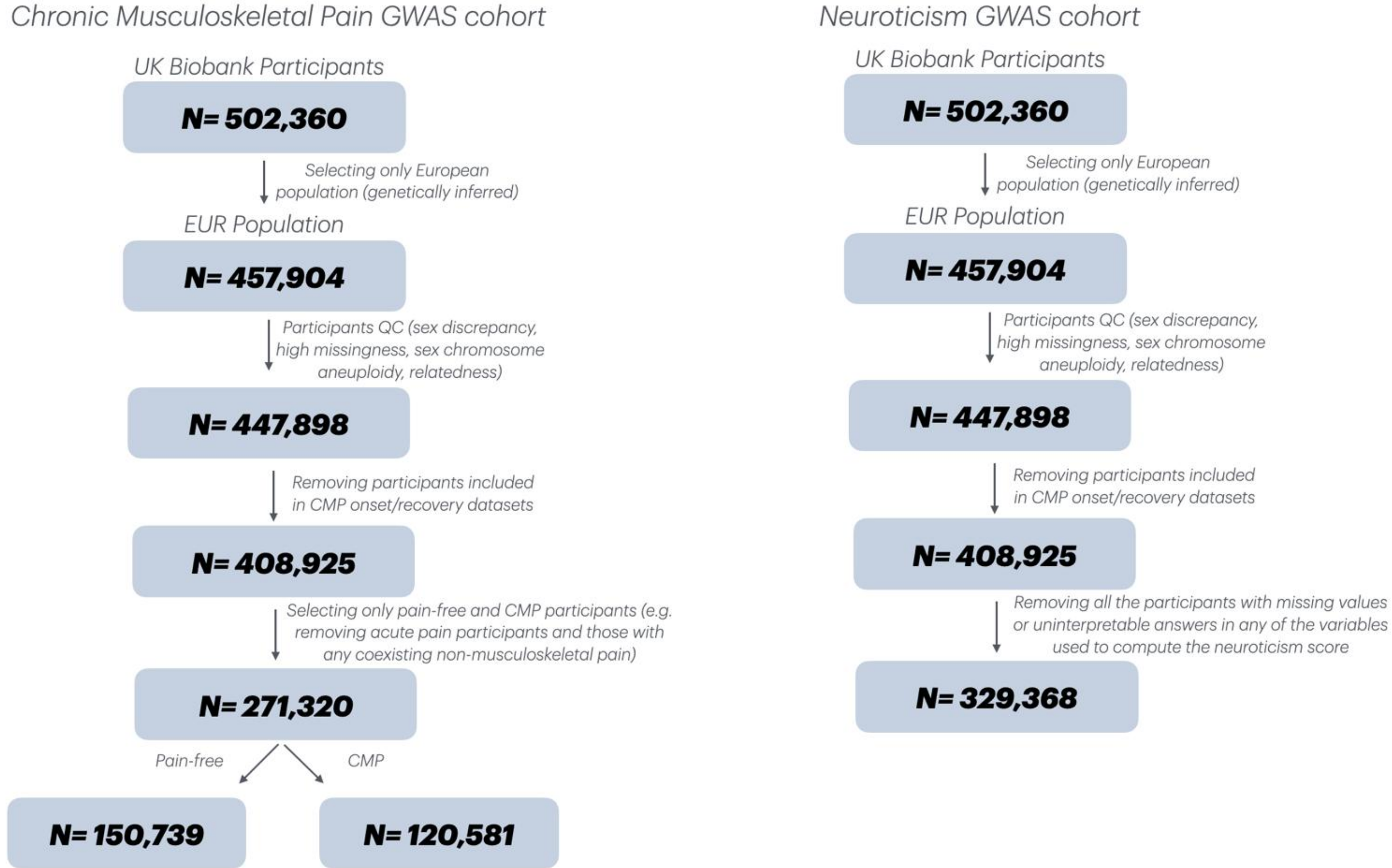

Figure S5. Flowchart of the GWAS cohort construction. Both GWAS were conducted only on participants with European ancestry. Quality control procedures were applied to remove participants with sex discrepancy (between self-reported and genetically inferred sex), high missingness, sex chromosome aneuploidy, and relatedness. In the CMP GWAS cohort, pain-free and CMP participants were extracted, excluding those with acute pain or coexisting non-musculoskeletal pain (headaches, general pain, facial pain, and abdominal pain). In the neuroticism GWAS cohort, participants with missing values or uninterpretable answers for any variable used to compute the neuroticism score were excluded.

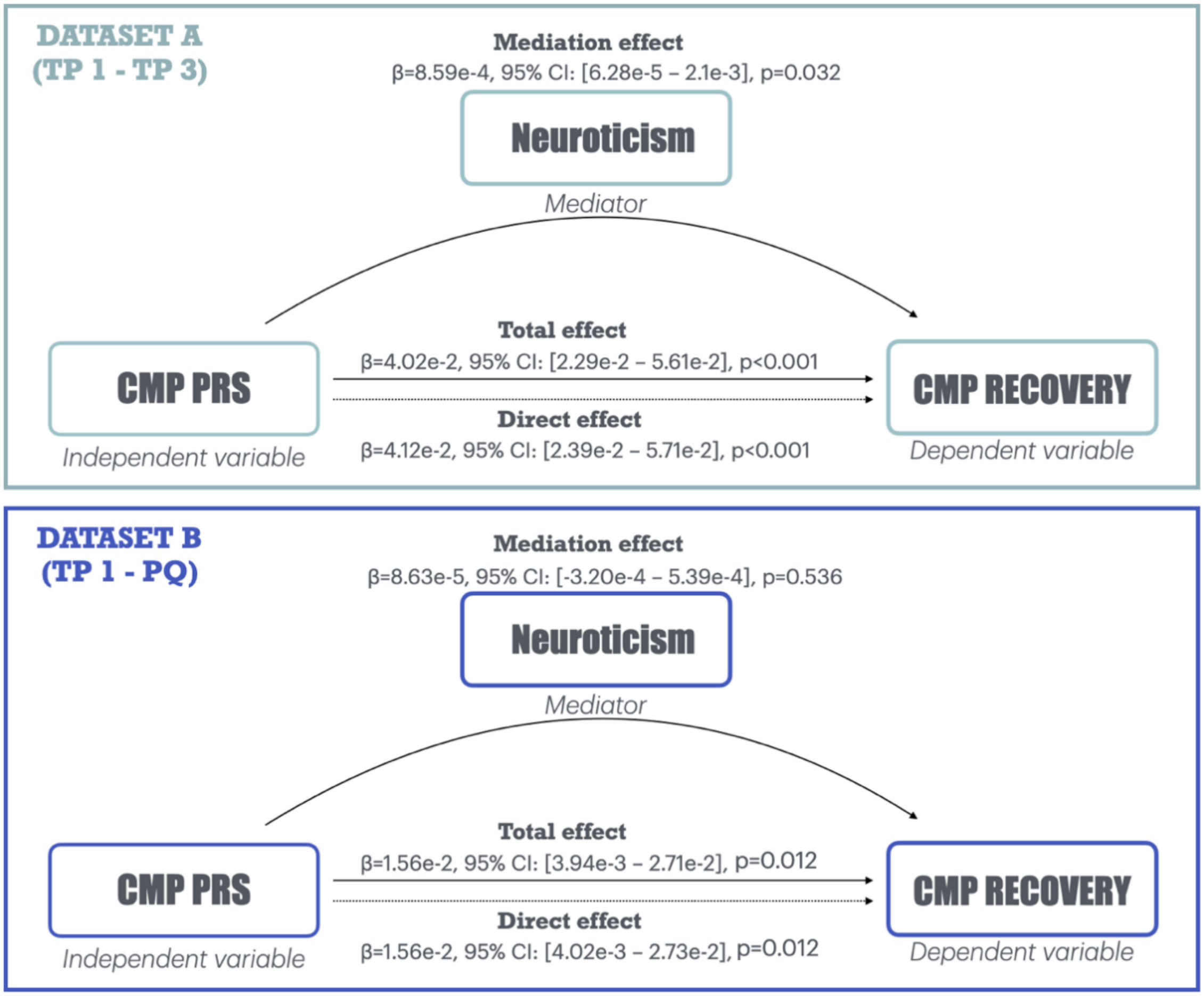

Figure S6. Results of the mediation analysis using the chronic musculoskeletal pain (CMP) recovery Datasets A-B. Neuroticism served as the mediator of the association between the CMP polygenic risk score (PRS), which was used as the independent variable, and the binary outcome (steady pain-free vs. CMP recovery) in Dataset A (TP 1 – TP 3). These results were not replicated in Dataset B (TP 1 – PQ).
